# Loss of TET function in T regulatory cells yields ex-Treg cells biased toward T follicular helper cells, causing autoimmune diseases through autoantibody production

**DOI:** 10.3389/fimmu.2026.1684023

**Published:** 2026-03-27

**Authors:** Kazumasa Suzuki, Leo J. Arteaga-Vazquez, Bruno Villalobos Reveles, Lot Hernández-Espinosa, Isaac F. López-Moyado, Atsushi Onodera, Daniela Samaniego-Castruita, Ferhat Ay, Arlet Lara-Custodio, Patrick G. Hogan, Hugo Sepulveda, Anjana Rao

**Affiliations:** 1 Center for Autoimmunity and Inflammation, La Jolla Institute for Immunology, La Jolla, CA, United States; 2 Center for Cancer Immunotherapy, La Jolla Institute for Immunology, La Jolla, CA, United States; 3 Moores Cancer Center, University of California (UC) San Diego, San Diego, CA, United States; 4 Department of Pharmacology, University of California (UC) San Diego, San Diego, CA, United States; 5 Sanford Consortium for Regenerative Medicine, La Jolla, CA, United States; 6 Institute for Advanced Academic Research (IAAR), Chiba University, Chiba, Japan; 7 Research Institute of Disaster Medicine (RIDM), Chiba University, Chiba, Japan; 8 Center for Human Immunological Diseases and Therapy Development (cCHID), Chiba University, Chiba, Japan; 9 Department of Pediatrics, University of California (UC) San Diego, San Diego, CA, United States; 10 Program in Immunology, University of California (UC) San Diego, San Diego, CA, United States; 11 Laboratory of Transcription and Epigenetics, Institute of Biomedical Sciences, Universidad Andres Bello, Santiago, Chile

**Keywords:** autoimmune diseases, DNA methylation, ex-Treg cells, FOXP3, inflammation, regulatory T(Treg) cell, TET methylcytosine dioxygenase, TET2 and TET3

## Abstract

**Introduction:**

T regulatory cells (Treg cells) express the transcription factor FOXP3 and maintain immune homeostasis by attenuating effector responses. Treg cells are prone to lose FOXP3 and convert to pathological ‘ex-Treg’ cells under conditions of strong or chronic inflammation. One mechanism for loss of FOXP3 expression involves increased DNA methylation of intronic enhancers *CNS1* and *CNS2* in the *Foxp3* locus; these enhancers are maintained in a demethylated state by TET enzymes, 5-methylcytosine (5mC) dioxygenases that generate 5-hydroxymethylcytosine (5hmC) and other oxidized methylcytosines that are essential intermediates in all pathways of DNA demethylation. We previously showed that FOXP3_+_ Treg cells from *Tet2/3*-deficient (*Tet2/3 DKO*) mice displayed increased methylation of *CNS1* and *CNS2* and converted to FOXP3-negative ex-Treg cells considerably more efficiently than wild-type (WT) Treg cells.

**Method:**

We extend our previous analysis of *Foxp3-Cre Tet2/3*_fl/fl_ mice.

**Results and discussion:**

We classified the mice as DKO-moderate or DKO-severe based on the total number of leukocytes in the spleen and peripheral lymph nodes and investigated the phenotypic and molecular basis for the progressive inflammation occurring in these mice. RNA-seq as well as histological and immunocytochemical analyses showed a striking expansion of T follicular helper (Tfh) cells and plasma cells in *Tet2/3 DKO*-severe mice. And single-cell (sc) RNA-seq analyses suggested that this was due to skewed differentiation of both *Tet2/3 DKO* FOXP3_+_ Treg cells and *Tet2/3 DKO FOXP3*_–_ ex-Treg cells into Tfh-like cells. Base-resolution “6-base” sequencing showed the expected loss of 5hmC and increased 5mC in Tfh cells purified from *Tet2/3 DKO*-severe mice, and suggested that the observed bias in gene expression patterns could arise either from a direct increase in methylation of essential enhancers due to TET deficiency, or from interference with binding of methylation-sensitive transcriptional repressors including CCCTC-bindingfactor (CTCF).

## Introduction

T regulatory cells (Treg cells) maintain immune homeostasis and immunological self-tolerance and suppress pathological inflammation in both humans and mice (1–8). The lineage-determining transcription factor FOXP3 controls the development and suppressive function of Treg cells (9, 10). FOXP3-expressing Treg cells are typically stable under steady state conditions *in vivo* (11) but can lose FOXP3 and convert to pathological cells called “ex-Treg” cells in strong or chronic inflammatory environments (11–20). Ex-Treg cells lose the ability to suppress inflammation and can additionally convert to effector CD4^+^ T cells of different lineages—Th1, Th2, Th17, and T follicular helper (Tfh) cells that secrete pro-inflammatory cytokines (IFN-γ, IL-4, IL-17, and IL-21)—thereby promoting chronic inflammation and the development of autoimmune/inflammatory diseases (11–20). Ex-Treg cells have been suggested to promote autoimmune reactions in several mouse models of disease, including type 1 diabetes (14), graft-versus-host disease (16), multiple sclerosis (17), and autoimmune arthritis (18, 19). However, it is still unclear how ex-Treg cells acquire inflammatory functions after loss of FOXP3.

The stability of FOXP3 expression during mouse Treg cell differentiation is regulated by two conserved non-coding sequence elements, *CNS1* and *CNS2*, located in the first intron of the *Foxp3* gene (5′ of the first coding exon) (21–23). *CNS2*, also termed Treg-specific demethylated region (TSDR) (24), controls the stability of *Foxp3* expression in a manner linked to the DNA modification status of *CNS2* (24–27). CpG sites in the *Foxp3CNS2* element are predominantly unmethylated (C/5fC/5caC) in Treg cells, but fully methylated (5mC/5hmC) in naïve T cells (21, 24, 27–32). The TET dioxygenases TET2 and TET3 act redundantly to deposit 5hmC at the *CNS1* and *CNS2* enhancers during Treg cell differentiation, resulting in their demethylation (loss of 5mC) (28, 33). The combined disruption of the *Tet2* and *Tet3* genes in developing thymocytes of *CD4-Cre Tet2/3^floxed/floxed^* (*Tet2/3^fl/fl^*) mice resulted in the striking antigen-dependent expansion of iNKT cells, together with massive inflammation due to partial loss of T regulatory cells in the thymus and their almost complete loss in the periphery (34). The loss of Treg cells in these *CD4-Cre Tet2/3^fl/fl^* mice was at least partly due to reduced Treg stability due to loss of FOXP3 expression, which in turn was due to increased DNA methylation of *CNS1* and *CNS2* (28, 30, 33, 35). The addition of the TET activator vitamin C during *in vitro* differentiation of mouse and human iTreg cells increased TET enzymatic activity, maintained the *CNS1* and *CNS2* enhancers in a demethylated state, and increased the stability of FOXP3 expression (28, 31). Importantly, both *CNS2*-deficient and *Tet2/3*-deficient Treg cells lose FOXP3 in a manner that depends on cell division (21, 22, 28). The loss of FOXP3 or *CNS2* could be ameliorated by treatment with IL-2 or certain other cytokines (22) or the combination of IL-2, retinoic acid, and vitamin C (28, 36).

To understand the specific role of TET2/TET3 in Treg cells *in vivo*, we examined *Foxp3-Cre Tet2/3^fl/fl^* mice. These mice developed a progressive autoimmune/inflammatory disease that was fatal by 8–22 weeks of age (33, 35). Treg cells from these mice displayed increased methylation of the intronic enhancers *CNS1* and *CNS2* in the *Foxp3* gene, albeit to a lesser extent than observed in *CD4-Cre Tet2/3^fl/fl^* mice, reflecting the fact that CD4 is expressed earlier than FOXP3 during Treg development. In this early study, we analyzed the molecular phenotypes of only two 14-week-old *Foxp3-Cre Tet2/3^fl/fl^* mice, and RNA-seq data from CD4^+^ FOXP3^−^ T cells from one of these two mice showed the upregulation of Tfh-related and Th17-related genes (33). Treg cells from *Foxp3Cre Tet2/3^fl/fl^* mice were more prone to lose FOXP3 expression and become “ex-Treg” cells compared to wild-type (WT) Treg cells (33). Notably, total CD4^+^ T cells from *Foxp3-Cre Tet2/3^fl/fl^* mice were uniformly able to elicit a dominant inflammatory disease after transfer into recipient immunocompetent mice (33), despite the presence of wild-type Treg cells in the recipient mice. The data emphasized two points: first, TET proteins are essential for the maintenance of Treg cell stability and immune homeostasis in mice, and second, the inflammatory phenotype in the transfer experiments was driven, at least in part, by ex-Treg cells that acquired effector function (33).

In this study, we expanded our analysis of the role of TET dioxygenases in Treg and ex-Treg cells from *Foxp3-CreTet2/3^fl/fl^* mice. Phenotypically, the mice showed clear evidence of splenomegaly and lymphadenopathy that we classified as moderate or severe based on the total number of leukocytes in the spleen. The frequencies and numbers of total CD4^+^ T cells and CD4^+^ FOXP3^+^ Treg cells increased strikingly in DKO-severe compared to WT mice. As the disease advanced from DKO-moderate to DKO-severe, the mice showed increased T-cell activation as judged by a progressive decrease in the numbers of naïve CD4^+^ T cells. Histological and immunocytochemical analyses, together with flow cytometry, showed increased lymphocyte infiltration into many organs, increased numbers of neutrophils and plasma cells in DKO-severe mice, and an expansion of cytotoxic CD8^+^ T cells with increased expression of perforin and granzyme B. By performing bulk and single-cell (sc) RNA-seq on CD4^+^ T cells from WT and DKO-severe mice, we found increased expression of Tfh-associated genes, which was confirmed using flow cytometry, revealing increased expression of Tfh surface markers in both FOXP3^+^ Treg cells and FOXP3^−^ ex-Treg cells of *Foxp3Cre DKO* mice. Changes in 5mC and 5hmC distribution presented a complex picture depending on the genes involved, suggesting that increased methylation due to TET deficiency could reflect direct methylation of essential enhancers or decreased binding of transcriptional repressors such as CTCF. We conclude that FOXP3-negative ex-Treg cells from mice with selective deficiency in *Tet2* and *Tet3* in Treg cells show biased conversion to Tfh cells and cause systemic inflammation with variable onset whose severity progressively increases with age.

## Results

### Defining the level of systemic inflammation in *Foxp3-CreTet2/3^fl/fl^* mice

**Figure 1:**
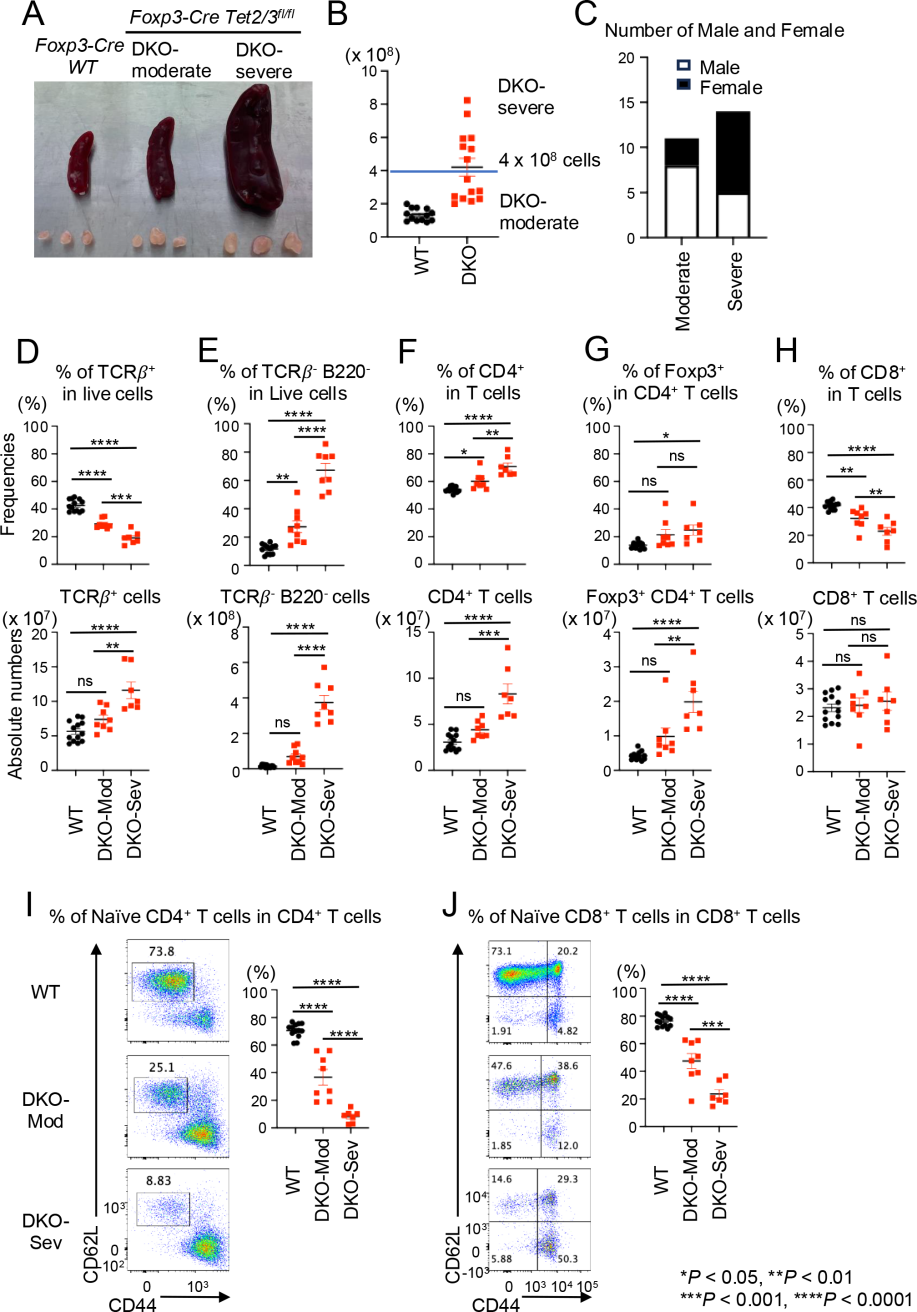
DKO-severe *Foxp3-CreTet2/3^fl/fl^* mice display CD4^+^ cells and TCRβ^−^ B220^−^ cells proliferation with strong inflammation. **(A)** Representative pictures of spleen and peripheral lymph nodes (cervical and inguinal) from *Foxp3Cre WT*, DKO-moderate, and DKO-severe *Foxp3-Cre Tet2/3^fl/fl^* mice (14-week-old). **(B)** Absolute cell number of pooled spleen and peripheral lymph nodes (cervical and inguinal) of *Foxp3Cre WT*, DKO-moderate, and DKO-severe *Foxp3-Cre Tet2/3^fl/fl^* mice (14-week-old). **(C)** Number of male and female in DKO-moderate and DKO-severe *Foxp3-Cre Tet2/3^fl/fl^* mice (14-week-old). (**D–H**) Quantification of the frequency (*top*) and absolute number (*bottom*) in pooled spleen and peripheral lymph nodes (cervical and inguinal) from *Foxp3Cre WT*, *Foxp3-Cre Tet2/3^fl/fl^* mice (14-week-old). **(D)** Frequency of TCRβ^+^ cells in live cells and absolute number. **(E)** Frequency of TCRβ^−^ B220^−^ cells in live cells and absolute number. **(F)** Frequency of CD4^+^ cells in TCRβ^+^ cells and absolute number. **(G)** Frequency of YFP(FOXP3)^+^ cells in TCRβ^+^ CD4^+^ cells and absolute number. **(H)** Frequency of CD8^+^ cells in TCRβ^+^ cells and absolute number. **(I, J)** Representative flow cytometry plots of CD62L^high^ CD44^low^ cells in TCRβ^+^ CD4^+^ cells **(I)** or TCRβ^+^ CD8^+^ cells **(J)** and quantification of the frequency of the cells in pooled spleen and peripheral lymph nodes (cervical and inguinal) from *Foxp3Cre WT*, *Foxp3-Cre Tet2/3^fl/fl^* mice (14-week-old). Error bars show mean ± SEM from at least three independent experiments. Statistical analysis was performed using one-way ANOVA (*p < 0.05, **p < 0.01, ***p < 0.001, and ****p < 0.0001).

At 14 weeks of age, *Foxp3-CreTet2/3^fl/fl^* mice could be divided into two groups distinguished by moderate or severe splenomegaly and lymphadenopathy (**Figure 1A**), defined as DKO-moderate and DKO-severe depending on whether they had fewer than or more than 4 × 10^8^ splenocytes and peripheral lymphocytes (from cervical and inguinal) (**Figure 1B**). The DKO-severe group contained a higher proportion of female mice (9/14, 64%) compared to the DKO-moderate group (3/11, 27%) (**Figure 1C**), suggesting that female mice are more likely to develop severe symptoms in *Foxp3-CreTet2/3^fl/fl^* mice. This conclusion was corroborated by the survival curve in the previous paper (reproduced here as **Supplementary Figure 1A**). No mice survive beyond early adulthood (6 months), indicating that the variability in disease development (the difference between moderate and severe phenotypes) is due to a variable time course of disease development in individual mice.

Although the percentage of T cells (TCRβ^+^ cells) among live cells in the spleen and lymph nodes decreased, the absolute number of T cells increased in DKO-severe compared to WT or DKO-moderate mice (**Figure 1D**). This reflected an increase in the frequencies and absolute numbers of cells other than T or B cells (TCRβ^−^ B220^−^ cells) in both DKO-moderate and DKO-severe mice (**Figure 1E**). CD4^+^ T-cell frequencies and absolute numbers increased slightly in the DKO-moderate group and considerably in the DKO-severe group compared to WT (**Figure 1F**). The absolute numbers of *Tet2/3DKO* Treg cells also increased, especially in DKO-severe mice (**Figure 1G**), but the levels of FOXP3 expression assessed using flow cytometry were similar to those of WT Treg cells (data not shown). These findings support our prior conclusion that Tregs in *Foxp3CreTet2/3^fl/fl^* mice have largely lost suppressive function and gained a dominant inflammatory phenotype, measured by their decreased ability, compared to wild-type Tregs, to correct the inflammation induced by *scurfy* cells in T-cell and bone marrow adoptive transfer experiments (33). The absolute numbers of CD8^+^ cells (as a fraction of all T cells) did not change in the spleen and lymph nodes of DKO-moderate or DKO-severe compared to WT mice (**Figure 1H**); however, the percentage of naïve cells (CD62L^high^ CD44^lo^) was dramatically decreased in both CD4^+^ and CD8^+^ T-cell populations in *Foxp3-CreTet2/3^fl/fl^* mice, especially DKO-severe mice, indicating that the majority of the T cells were activated or had become memory cells (**Figures 1I, J**).

### Apparent skewing of *Tet2/3 DKO* CD4^+^ YFP(FOXP3)^−^ ex-Treg cells toward Tfh- and Th17-like cells

We performed RNA sequencing on CD4^+^ YFP(FOXP3)^−^ T cells from WT, DKO-moderate, or DKO-severe mice. WT CD4^+^ YFP(FOXP)^−^ T cells, which contained ~70% naïve T cells (**Figures 1I**, top), clustered relatively closely (less than 9% variance in a Principal Component Analysis (PCA) plot, **Supplementary Figure 1B**), whereas CD4^+^ YFP(FOXP)^−^ T cells from DKO-moderate and DKO-severe *Foxp3-CreTet2/3^fl/fl^* mice showed more variability—likely because they are a heterogeneous cell population containing i) *Tet2/3DKO* ex-Treg cells arising from loss of FOXP3 stability and ii) conventional (non-Treg) CD4^+^ T cells mostly displaying an activated or memory phenotype (**Figure 1I**, middle and bottom panels). The DKO-moderate and DKO-severe CD4^+^ T cells showed a significant upregulation of Tfh-related genes (*Bcl6*, *Cxcr5*, *Pdcd1*, *Tox2*, *Maf*, *Batf*, *Ascl2*, *Il21*, *Il4*, *Klrb1c*, and *Nfkb1*), Th17-related genes (*Rorc*, *Il17a*, and *Il17f*), and cell cycle genes (*Cdkn1a*, *Cdkn2a*, *E2f7*, and *E2f8*) (data not shown) compared to WT T cells (MA plots, **Supplementary Figures 1C, D**), suggesting that a proportion of *Tet2/3 DKO* Treg cells had converted into proliferating ex-Treg cells with Tfh-like or Th17-like phenotypes.

**Figure 2:**
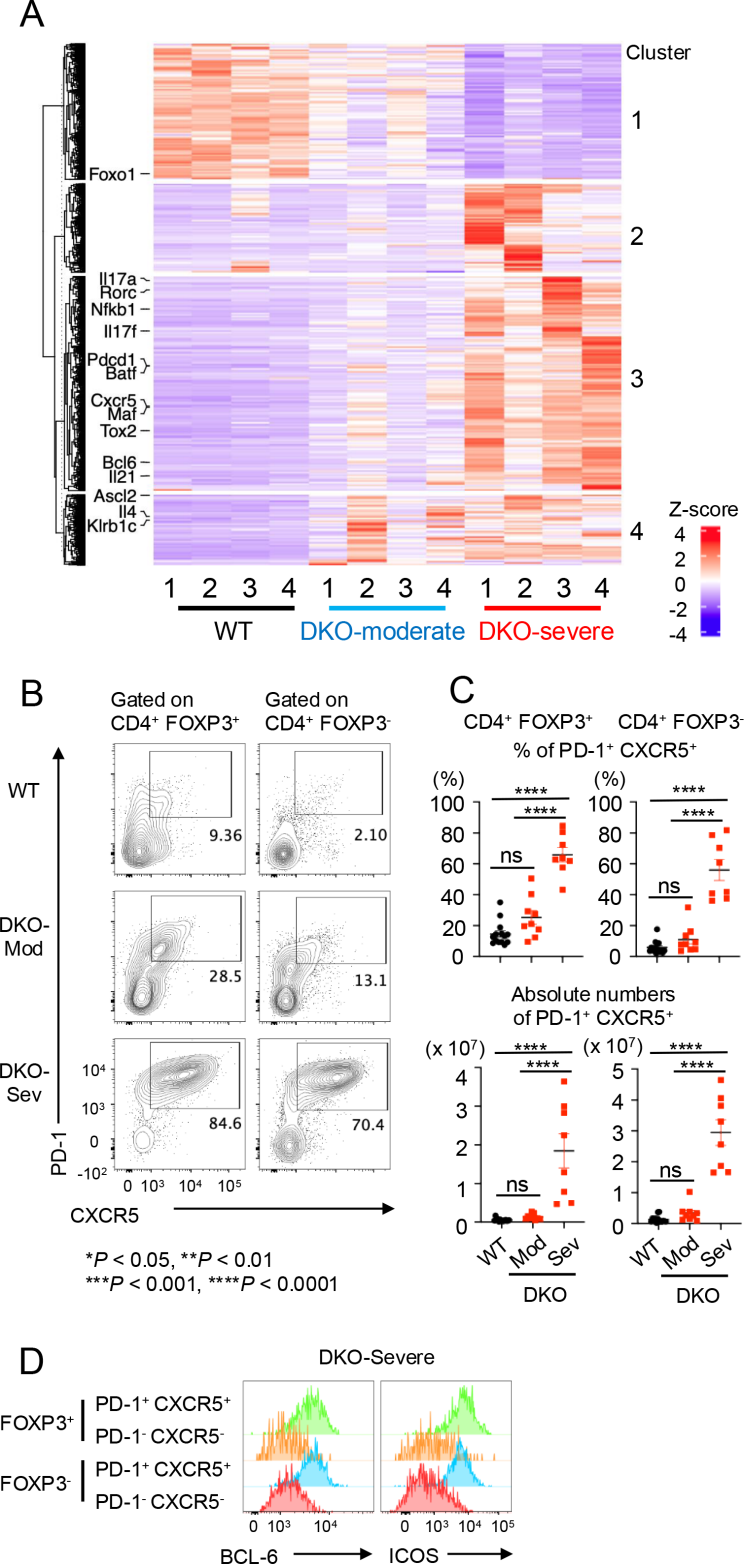
Tfh-like cells expanded in DKO-severe *Foxp3-CreTet2/3^fl/fl^* mice in bulk RNA sequencing and flow cytometry analysis. **(A)** Heatmap for the expression of clustered differentially expressed genes (DEGs). CD4^+^ YFP(FOXP3)^−^ T cells were sorted from pooled spleen and peripheral lymph nodes (cervical and inguinal) from *Foxp3Cre WT* mice and *Foxp3-Cre Tet2/3^fl/fl^* mice (14-week-old), and RNA sequencing was performed (n = 4). DEGs were extracted and clustered. Heatmap for the expression of clustered DEGs was generated. Tfh-related (*Bcl6*, *Cxcr5*, *Pdcd1*, *Tox2*, *Maf*, *Batf*, *Nfkb1*, *Ascl2*, *Il21*, *Il4*, *Klrb1C*, and *Foxo1*) and Th17-related (*Rorc*, *Il17a*, and *Il17f*) genes were indicated. **(B)** Flow cytometry analysis of PD-1^+^ CXCR5^+^ cells [gated on TCRβ^+^ CD4^+^ YFP(FOXP3)^+^ or TCRβ^+^ CD4^+^ YFP(FOXP3)^−^ cells] in pooled spleen and peripheral lymph nodes (cervical and inguinal) from 13–15-week-old *Foxp3Cre WT* and *Foxp3-Cre Tet2/3^fl/fl^* mice. **(C)** Quantification of the frequency of PD-1^+^ CXCR5^+^ cells in TCRβ^+^ CD4^+^ YFP(FOXP3)^+^ or TCRβ^+^ CD4^+^ YFP(FOXP3)^−^ cells and absolute number in pooled spleen and peripheral lymph nodes (cervical and inguinal). Error bars show mean ± SEM from at least three independent experiments. Statistical analysis was performed using one-way ANOVA (*p < 0.05, **p < 0.01, ***p < 0.001, and ****p < 0.0001). **(D)** Flow cytometry analysis of BCL6^+^ or ICOS^+^ cells (gated on PD-1^+^, CXCR5^+^ or PD-1^−^, CXCR5^−^ cells in FOXP3^+^ or FOXP3^−^ CD4^+^ T cells) in pooled spleen and peripheral lymph nodes (cervical and inguinal) from 13–15-week-old DKO-severe mice. "ns" is "not significant".

When differentially expressed genes were extracted and clustered into a heatmap (**Figure 2A**), most Tfh-related (*Bcl6*, *Cxcr5*, *Pdcd1*, *Tox2*, *Maf*, *Batf*, *Il21*, and *Nfkb1*) and Th17-related (*Rorc*, *Il17a*, and *Il17f*) genes were expressed more strongly in DKO-severe than in DKO-moderate or WT CD4^+^ T cells (heatmap cluster 3). In contrast, *Foxo1*—a suppressor of Tfh differentiation—was less highly expressed in DKO cells compared to WT (heatmap cluster 1). These results suggested that loss of FOXP3 expression in *Tet2/3 DKO* ex-Treg cells may result in expansion of ex-Treg cells with gene expression patterns resembling those of Tfh or Th17 cells.

A relatively small and variable proportion of cells in total splenic CD4^+^ T cells of DKO-severe mice produced IL-17A upon phorbol 12-myristate 13-acetate (PMA)/ionomycin stimulation (1%–12%, average ~5%; **Supplementary Figures 2A**, left, **2D**). In contrast, we observed a striking and progressive expansion of Tfh-like PD-1^+^ CXCR5^+^ cells in DKO-severe mice via flow cytometry of both residual CD4^+^ YFP(FOXP3)^+^ Treg cells and CD4^+^ YFP(FOXP3)^−^ cells (containing expanded ex-Treg cells). A remarkable proportion (55%–65%) of both CD4^+^ FOXP3^+^ and CD4^+^ FOXP3^−^ cells in DKO-severe mice were PD-1^+^ CXCR5^+^ Tfh-like cells (range 45%–85%, **Figures 2B, C**). Some of these cells may be derived from expanded ex-Treg cells that lost FOXP3 and acquired skewed gene expression patterns that resemble those of PD-1^+^ CXCR5^+^ Tfh-like cells, and others from bystander CD4^+^ T cells due to the systemic inflammation developing in mice with Treg-specific *Tet2/Tet3* deletion. In DKO-severe mice, PD-1^+^ CXCR5^+^ cells showed higher expression of the Tfh cell markers BCL6 and ICOS compared with non-Tfh (PD-1^-^ CXCR5^-^) cells (**Figure 2D**); consistent with the increase in Tfh-like cells, we also observed a significant increase in the frequency of Fas^+^ GL-7^+^ GC B cells in B220^+^ cells in the spleens of DKO-severe compared to DKO-moderate or WT mice (**Supplementary Figure 2K**), even though the frequencies of total B220^+^ cells declined (**Supplementary Figure 2J**). We also noted a substantial increase in the frequencies of bystander CD8^+^ T cells that produced IFN-γ upon PMA/ionomycin stimulation, and CD8^+^ T cells with increased perforin and granzyme B expression, in DKO-severe compared to WT mice (**Supplementary Figures 2B, C, F–H**), even though CD8^+^ T cells showed no overall expansion (**Figure 1H**). The proportion of IL-21-secreting CD4^+^ T cells after PMA/ionomycin stimulation was markedly increased in DKO-severe mice (**Supplementary Figure 2I**).

### Greater expansion of Tfh-like cells compared to Th17-like cells in DKO-severe *Foxp3-CreTet2/3^fl/fl^* mice

**Figure 3:**
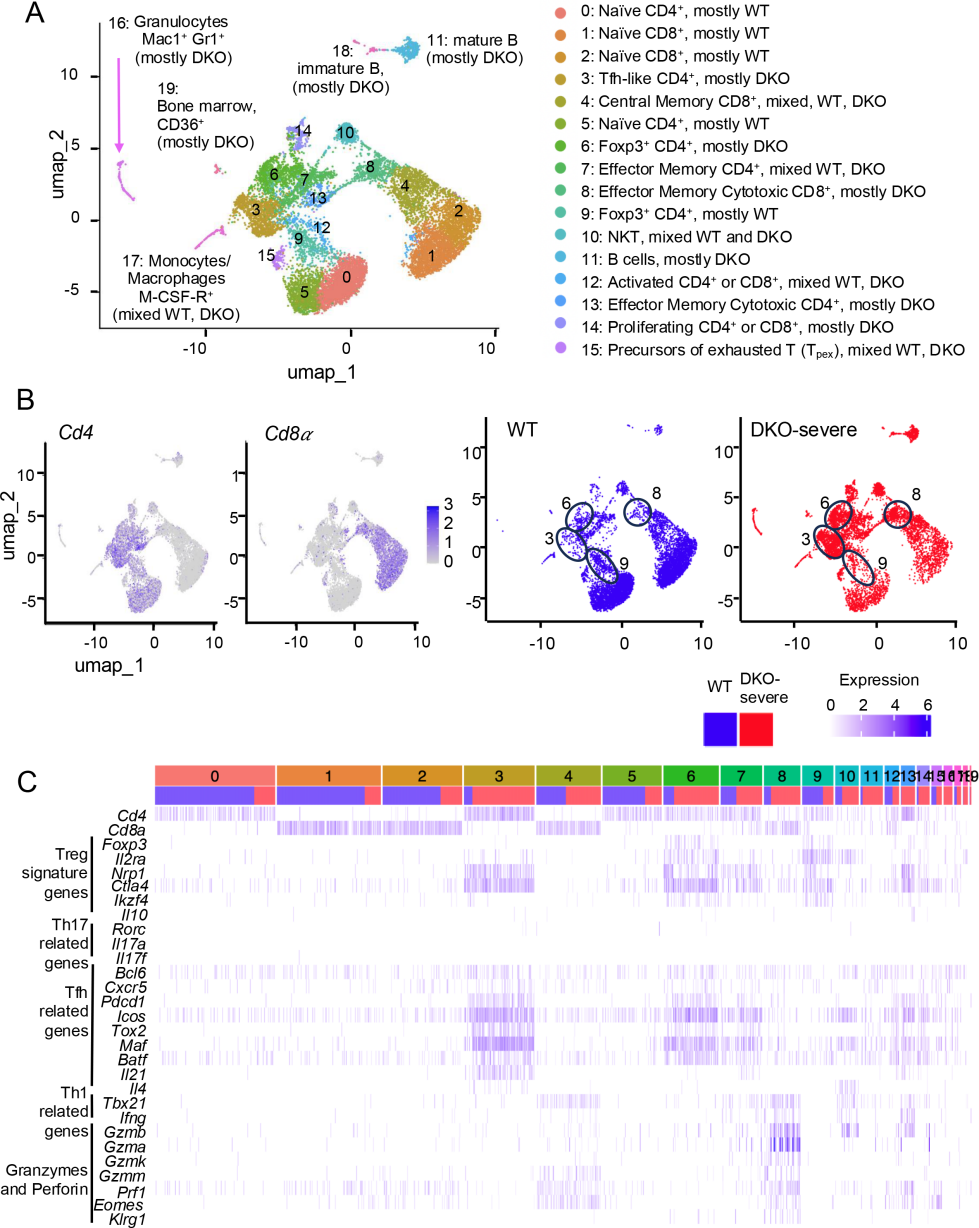
Tfh-like cells expanded in DKO-severe *Foxp3-CreTet2/3^fl/fl^* mice at the single-cell level analysis. **(A–C)** Single-cell (sc) RNA sequencing for T cells from WT or DKO-severe mice. TCRβ^+^ cells were sorted from pooled spleen and peripheral lymph nodes (cervical and inguinal) from *Foxp3Cre WT* mice and *DKO-severe Foxp3-Cre Tet2/3^fl/fl^* mice (14-week-old), and scRNA sequencing was performed (n = 1). **(A, B)** The UMAP plot shows 0–19 clusters (**A**, *left*). Annotated clusters are shown. Expression of *Cd4* and *Cd8α* (**B**, *left two*) and distribution of WT (Blue) and *Tet2/3DKO* (Red) population (**B**, *right two*). **(C)** Expression map of indicated genes. Blue column, cells from WT mice; red column, cells from DKO-severe mouse.

To determine whether *Tet2/3 DKO* ex-Treg cells skew mainly to Tfh or Th17 cells and to address the impact of *Tet2/3 DKO* ex-Treg cell expansion on bystander CD4^+^ or CD8^+^ T cells, we compared patterns of gene expression at the single-cell level in WT and DKO-severe TCRβ^+^ T cells (**Figure 3**). Single-cell RNA sequencing (scRNA-seq) distinguished 19 clusters in Uniform Manifold Approximation and Projection (UMAP) analysis (**Figure 3A**). *Cd4^+^* and *Cd8^+^* cells occupy the *left* and *right* arms of the UMAP plot, respectively (**Figure 3B**, left). Cells in clusters 0 and 5 were primarily WT naïve CD4^+^, cells in clusters 1 and 2 were primarily WT naïve CD8^+^ T cells, and cells in clusters 6 (mostly DKO) and 9 (mostly WT) were Treg cells based on the expression of *Cd4* and *Foxp3* (**Figure 3B** *right*, **Figure 3C**); by flow cytometry, the WT and DKO FOXP3^+^ Treg cells expressed similar levels of CD4 and FOXP3 (data not shown). Interestingly, *Tet2/3 DKOFoxp3*^+^ Treg cells (Cluster 6) expressed not only *Foxp3* and other Treg signature genes (*Il2ra*, *Nrp1*, *Ctla4*, and *Ikzf4*) but also Tfh-related genes (*Bcl6*, *Cxcr5*, *Pdcd1*, *Icos*, *Tox2*, *Maf*, and *Batf*), suggesting that conversion to Tfh-like ex-Treg cells was initiated in *Tet2/3 DKOFoxp3*^+^ Treg cells prior to the loss of FOXP3 (**Figure 3C**). Indeed, many FOXP3^+^ CD4^+^ cells in DKO-severe mice express Tfh markers (**Figures 2B, D**), suggesting that the DNA methylation changes in Tfh-related genes that initiate Treg to Tfh skewing can occur earlier than the loss of FOXP3 protein detected using flow cytometry. The time dependence of Treg to Tfh skewing is illustrated by comparing the cells in cluster 6 (mostly *Foxp3*^+^ DKO) with those in cluster 3 (mostly Foxp3^−^ DKO), which expressed Tfh-related genes even more strongly (**Figure 3C**).

Although they were clearly detected by bulk RNA-seq of the entire DKO-severe cell population (**Figure 2A**; **Supplementary Figure 1D**), *Rorc* and *Il17a/Il17f* expression was barely detected in scRNA-seq of *Tet2/3 DKO* cells (**Figure 3C**), suggesting that Th17 cells were not a major population in *Tet2/3 DKO* ex-Treg cells. The percentages of IFN-γ^+^ cells (presumably Th1 cells) were decreased rather than increased in CD4^+^ cells from *Tet2/3 DKO*-severe mice (**Supplementary Figures 2A**, right, ** 2E**); in other models, ex-Treg cells have been reported to possess Th1 characteristics (12, 14, 17). CD8^+^ T cells showed increased cytotoxic function in *Tet2/3 DKO*-severe compared to WT or DKO-moderate mice, as judged via scRNA-seq for *Tbx21*, *Ifng*, *Granzymes* (*Gzma*, *Gzmb*, *Gzmk*, and *Gzmm*), and *Prf1* encoding perforin (**Figure 3C**, cluster 8 in scRNA-seq expression map), consistent with flow cytometry for IFN-γ, granzyme B, and perforin (**Supplementary Figures S2B, C, F–H**).

Because Foxp3Cre expression has been considered “leaky” (37, 38), we asked whether Tfh cells in *Foxp3-CreTet2/3^fl/fl^* mice expressed or had lost *Tet2*. We measured *Tet2* expression in Treg and Tfh cells from WT and *Foxp3-CreTet2/3^fl/fl^* mice via qPCR to determine whether Tfh cells in *Foxp3-CreTet2/3^fl/fl^* mice were derived from *Tet2/3*-deficient ex-Treg cells (**Supplementary Figures S3A, B**). Data from the DICE [Database of Immune Cell Expression, Expression quantitative trait loci (eQTLs) and Epigenomics] project showed little difference in *Tet2* and *Tet3* mRNA expression when comparing WT Tfh cells and other WT CD4^+^ T cells, including non-activated naïve, Th1, Th2, Th17, and Treg cells (https://dice-database.org/landing; **Supplementary Figure 3C**). Relative to WT and heterozygous *Tet2^+/−^* Treg cells, FOXP3^+^ *Tet2/3 DKO* Treg cells did not detectably express *Tet2* as expected (**Supplementary Figure 3A**), whereas Tfh cells from DKO-moderate and DKO-severe *Foxp3-CreTet2/3^fl/fl^* mice expressed significantly lower and undetectable levels of *Tet2*, respectively, compared to WT CD4^+^ non-Treg cells, CD4^+^ cells not expressing Tfh surface markers (non-Tfh), and naïve CD4^+^ cells (**Supplementary Figure 3B**). *Tet2* expression was particularly reduced in Tfh cells from DKO-severe mice (**Supplementary Figure 3B**), suggesting that almost all Tfh cells in DKO-severe *Foxp3-CreTet2/3^fl/fl^* mice were derived from *Tet2/3 DKO* ex-Treg cells, or that stochastic loss of *Tet2* in leaky *Foxp3*-deleted precursor cells also skewed their differentiation toward Tfh cells. In fact, analysis of total *Tet2/3-deficient* CD4^+^ T cells showed increased numbers of PD-1^+^ CXCR5^+^ Tfh cells (**Supplementary Figure 3D**). In each case, we predict that a transient decrease in TET activity may be needed for optimal Tfh development.

To confirm the (ex)-Treg origin of Tfh cells in *Foxp3-Cre Tet2/3^fl/fl^* mice, we performed fate-mapping studies. We analyzed the proportion of Tfh cells expressing YFP from the *Rosa26* locus using the *Foxp3-Cre Tet2/3^fl/fl^Rosa26-YFP^LSL^* mouse strain in which Cre^+^ Treg cells also express *Rosa26-YFP* from the *Rosa26* locus. Treg cells in this strain also express the YFP–Cre fusion protein encoded in the *Foxp3* gene; however, as described previously (33), cells expressing YFP from the *Rosa26* locus (expressed Treg and ex-Treg cells) can be easily distinguished by their high YFP intensity from those expressing the YFP–Cre fusion protein (expressed at lower intensity only in FOXP3^+^ Treg cells). We further stained cells with anti-FOXP3 to exclude Treg cells from the FOXP3^−^ Tfh cell analysis. In DKO-severe mice, almost all FOXP3^−^ Tfh cells (PD-1^+^ CXCR5^+^ in CD4^+^ FOXP3^-^ T cells) were Rosa26-YFP-expressing cells, indicating that they are ex-Treg cells (**Supplementary Figure 3E**). The proportion of Rosa26-YFP^+^ Tfh cells was reduced in heterozygous (*Tet2/3^fl/+^*) mice and DKO-moderate mice, indicating that the frequency of conversion to Tfh-like ex-Treg cells is low in heterozygous *Tet2/3^fl/+^* mice and increases as mice progress from the moderate to severe *Tet2/3^fl/f^ Foxp3Cre* DKO-severe phenotype (**Supplementary Figure 3E**).

### Expansion of plasma cells and Tfh cells in secondary lymphoid tissues of DKO-severe *Foxp3-CreTet2/3^fl/fl^* mice

**Figure 4:**
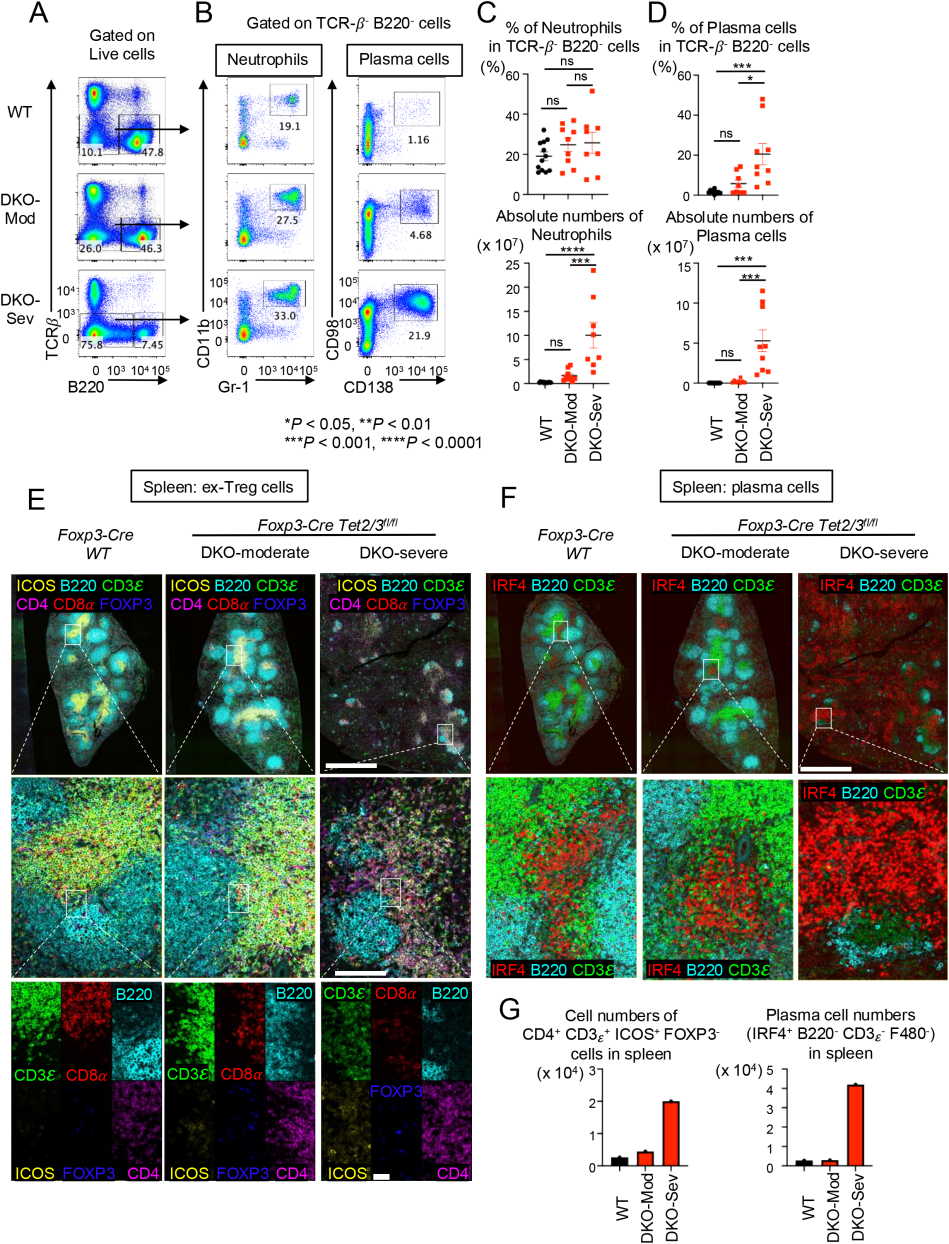
Along with proliferation of Tfh-like *Tet2/3 DKO* ex-Treg cells, plasma cells were diffusely expanded in spleen from DKO-severe mice. **(A)** Flow cytometry analysis of TCRβ^−^ B220^−^ cells (gated on live cells) in pooled spleen and peripheral lymph nodes (LNs) (cervical and inguinal) of 13–15-week-old *Foxp3Cre WT* and *Foxp3-Cre Tet2/3^fl/fl^* mice. **(B)** Flow cytometry analysis of neutrophils (CD11b^+^ Gr-1^+^ cells) or plasma cells (CD98^+^ CD138^+^ cells) (gated on TCRβ^−^ B220^−^ cells) in pooled spleen and peripheral LNs (cervical and inguinal) from 13–15-week-old *Foxp3Cre WT* and *Foxp3-Cre Tet2/3^fl/fl^* mice. **(C)** Quantification of the frequency of CD11b^+^ Gr-1^+^ cells in TCRβ^−^ B220^−^ cells and absolute number. **(D)** Quantification of the frequency of CD98^+^ CD138^+^ cells in TCRβ^−^ B220^−^ cells and absolute number. Error bars show mean ± SEM from at least three independent experiments. Statistical analysis was performed using one-way ANOVA (*p < 0.05, **p < 0.01, ***p < 0.001, and ****p < 0.0001). **(E)** Immunostaining of CD3ϵ, CD8α, B220, ICOS, FOXP3, and CD4 in spleen from 14-week-old *Foxp3Cre WT* and *Foxp3-Cre Tet2/3^fl/fl^* mice. Scale bars: top, 1 mm; middle, 100 μm; bottom, 25 μm. **(F)** Immunostaining of IRF4, B220, and CD3ϵ in spleen from 14-week-old *Foxp3Cre WT* and *Foxp3-Cre Tet2/3^fl/fl^* mice. Scale bars: top, 1 mm; bottom, 100 μm. **(G)** The graphs show the cell numbers of CD4^+^ CD3ϵ^+^ ICOS^+^ FOXP3^−^ cells or IRF4^+^ B220^−^ CD3ϵ^−^ F480^−^ cells counted in the stained sections from spleen. "ns" is "not significant".

As mentioned above, although the percentage of T cells (TCRβ^+^ cells) in live cells decreased, the absolute number of T cells increased in DKO-severe *Foxp3CreTet2/3^fl/fl^* mice (**Figure 1D**). Similarly, the most expanded cell populations in the spleens and peripheral lymph nodes of DKO-severe were a TCRβ^−^ B220^−^ population (~70%) (**Figure 4A**), which contained CD11b^+^ Gr-1^+^ neutrophils (**Figure 4B** *left*, **C**) as well as an expanded population of CD98^+^ CD138^+^ plasma cells (**Figures 4B** *right*, **4D**). We attribute plasma cell expansion in *Foxp3CreTet2/3^fl/fl^* mice to the Tfh activity of the expanded *Tet2/3 DKO* ex-Treg cells, which would be expected to promote B-cell activation in the germinal center and plasma cell differentiation.

We performed immunostaining analysis for Tfh-like *Tet2/3 DKO* ex-Treg cells and plasma cells. In **Figure 4E**, there are clear T-cell and B-cell regions (as well as germinal centers; data not shown) in the spleens from WT or DKO-moderate mice, but these structures were destroyed in DKO-severe mice (**Figure 4E**, top). In T cells of WT, the ratio of CD4^+^ T cells to CD8^+^ did not differ perceptibly, and there were almost no ICOS^+^ cells in WT spleens (**Figure 4E**, left middle and bottom panels); in contrast, the proportion of CD4^+^ cells and ICOS^+^ cells increased slightly in DKO-moderate and significantly in DKO-severe spleen (**Figure 4E**, right two panels, middle and bottom) in the T cell–B cell border region. ICOS is a well-established marker for Tfh cells; given that a substantial number of *Tet2/3 DKO* Treg cells in DKO-severe mice have not yet lost *Foxp3* but express *Icos* (cluster 6 in **Figure 3C**), a likely possibility is that the ICOS^+^ CD4^+^ FOXP3^−^ cells in *Foxp3CreTet2/3^fl/fl^* mice are derived from TET-deficient Treg cells that converted into Tfh-like ex-Treg cells that gain ICOS expression (see Discussion).

In **Figure 4F**, we used IRF4 as a plasma cell marker. IRF4^+^ cell clusters were observed in WT and DKO-moderate spleens, but their number and extent were limited (**Figure 4F**, left two panels). In contrast, IRF4-positive cells were observed extensively throughout the spleens of DKO-severe mice (**Figure 4F**, right panels), consistent with the increase in frequencies and absolute numbers of CD98^+^ CD138^+^ plasma cells in the spleens of these mice (**Figures 4B**, right, **D**). The absolute numbers of FOXP3^−^ Tfh-like cells and plasma cells in the spleen sections were also strikingly increased in *Tet2/3 DKO*-severe phenotype mice compared to WT mice (**Figure 4G**).

Finally, we tested the polyreactivity of serum isolated from WT (n = 8), DKO-moderate (n = 3), and DKO-severe mice (n = 4) by autoantibody array assay and observed elevated levels of autoantibodies related to autoimmune diseases (**Supplementary Figure 4**). The assay between groups was conducted using the R package “limma”, and multiple comparisons correction was performed (adjusted *p*-value < 0.05 between tested groups). Consistent with plasma cell expansion, the levels of many autoantibodies were elevated variably but substantially in DKO-severe compared to WT or DKO-moderate mice; different mice showed different patterns of autoantibody production (**Supplementary Figure 4**).

### Strong inflammation and autoimmune reactions in several tissues of DKO-severe *Foxp3-CreTet2/3^fl/fl^* mice

**Figure 5:**
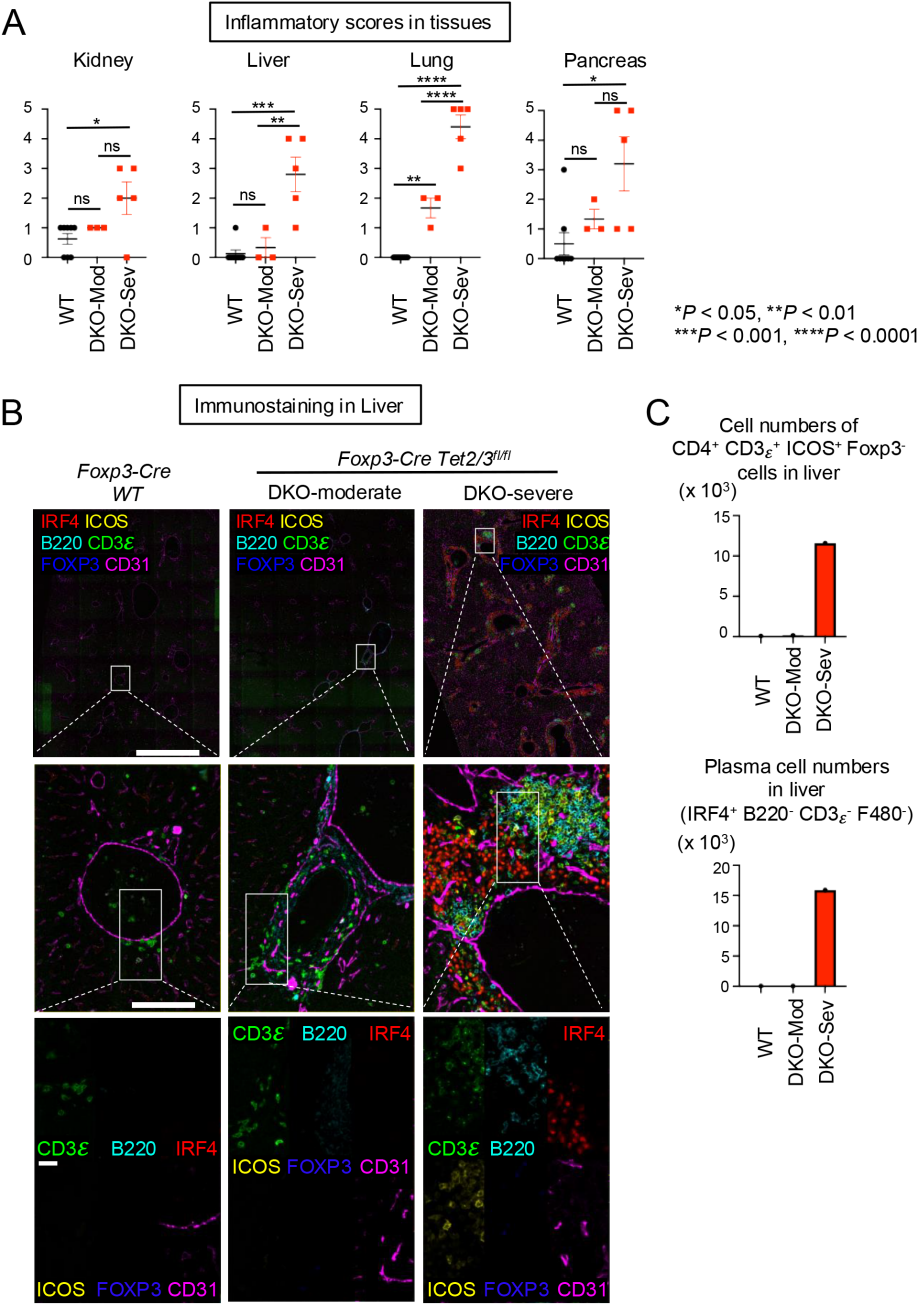
Systemic inflammation accompanied by infiltration of Tfh-like ex-Treg cells and plasma cells damaged organs in DKO-severe mice. **(A)** Inflammation scores (0–5) measured from H&E staining. Error bars show mean ± SEM from at least three independent experiments. Statistical analysis was performed using one-way ANOVA (*p < 0.05, **p < 0.01, ***p < 0.001, and ****p < 0.0001). **(B)** Immunostaining of CD3ϵ, B220, IRF4, ICOS, FOXP3, and CD31 in liver from 14-week-old *Foxp3Cre WT* and *Foxp3-Cre Tet2/3^fl/fl^* mice. Scale bars: top, 1 mm; middle, 100 μm; bottom, 25 μm. **(C)** The graphs show the cell numbers of CD4^+^ CD3ϵ^+^ ICOS^+^ Foxp3^−^ cells or IRF4^+^ B220^−^ CD3ϵ^−^ F480^−^ cells counted in the stained sections from liver. "ns" is "not significant".

We performed multi-tissue necroscopy analysis for WT, DKO-moderate, and DKO-severe mice to evaluate the degree of inflammation using our Inflammation score (0–5) for hematoxylin and eosin (H&E) staining samples from the pancreas, colon, skeletal muscle, heart, liver, lung, salivary gland, brain, knee joint, and stomach. Although we did not perform clinical pathological evaluations to assess tissue function, we observed strong inflammation and lymphocyte infiltration in the kidney, liver, lung, and pancreas of DKO-severe mice (**Figure 5A**; **Supplementary Figure 5**). Histologically, in the kidneys of WT and DKO-moderate mice, infiltrates were absent or limited to sparse lymphocytes and macrophages, whereas DKO-severe mice exhibited prominent mixed cortical interstitial infiltrates enriched in plasma cells, particularly surrounding renal veins near the fornix, which occasionally extended into the urothelium. While ulceration of the urothelium was not observed, infiltration of the epithelium could potentially disrupt epithelial barrier function. In the livers of DKO-severe mice, large numbers of lymphocytes, plasma cells, and macrophages expanded periportal regions and filled hepatic sinusoids. In severely affected livers, up to an estimated 20% of tissue sections were occupied by infiltrating cells. However, in partial hepatectomy experiments, ~75% of the mouse liver can be resected without functional impairment (39), suggesting that the mice may not have experienced hepatic insufficiency. In the lungs, DKO-moderate mice had perivascular and peribronchiolar mixed infiltrates dominated by lymphocytes, consistent with infiltrated inducible bronchus-associated lymphoid tissue (BALT), which were more extensive in DKO-severe mice, forming dense cuffs with increased plasma cell content. Interestingly, the presence of inducible BALT has not been shown to impede pulmonary function and, in other contexts, may improve pulmonary function by attenuating inflammation (40). In the pancreas, DKO-moderate mice occasionally exhibited lymphocytic infiltration of islets without statistical significance, which may, however, suggest some degree of endocrine pancreatic insufficiency (41). Pancreata from DKO-severe mice were densely infiltrated by plasma cells, lymphocytes, and macrophages; in the most affected cases, this immune infiltrate completely effaced normal pancreatic architecture (**Supplementary Figure 5**), virtually ensuring the presence of both endocrine and exocrine insufficiency (42) (**Supplementary Figure 5**).

To determine what cell types infiltrated perivascular regions in the livers from DKO-severe mice, we performed immunostaining of liver sections. The livers of WT and DKO-moderate mice showed infiltration of a few CD3ϵ^+^ cells (**Figure 5B**, left *two panels*) that did not express ICOS, suggesting that these T cells were not Tfh-like cells (**Figures 5B**, middle panels; **5C**, top). However, in perivascular regions in the livers from DKO-severe mice, CD3ϵϵ^+^ ICOS^+^ cells, B220^+^ cells, and IRF4^+^ cells were observed (**Figures 5B**, right bottom panel; **5C**); only a few or no *Tet2/3 DKO* CD3ϵ^+^ ICOS^+^ Tfh-like cells expressed FOXP3. The IRF4^+^ cells were plasma cells whose proliferation we had already noted in the spleen (**Figure 4**). Together, these results suggested that *Tet2/3 DKO* Tfh-like ex-Treg cells and plasma cells proliferated in secondary lymphoid tissues and migrated to peripheral tissues, possibly exacerbating inflammation directly.

### *Bcl6* upregulation with concomitant hypermethylation of the *Bcl6* locus in *Tet2/3 DKO* Tfh cells

**Figure 6:**
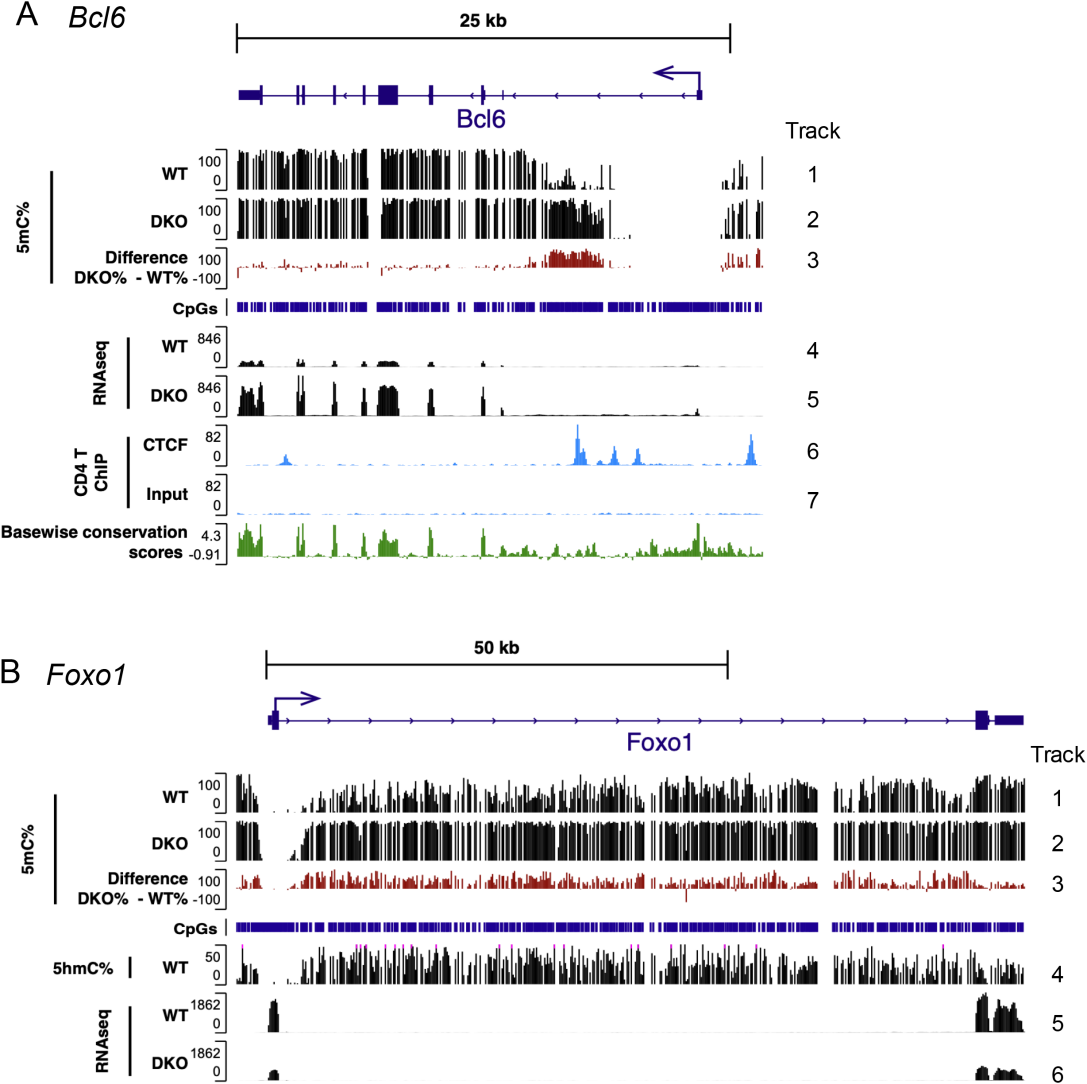
Characteristic hypermethylation accumulated in the first intron of the *Bcl6* locus in *Tet2/3DKO* Tfh cells. **(A)** Genome browser views showing 5mC% (tracks 1–3) from 6-base sequencing, gene expression (RNA-seq, tracks 4 and 5), CTCF binding (ChIP-seq, tracks 6 and 7) with CpGs, basewise conservation scores in *Bcl6* locus. **(B)** Genome browser views showing 5mC% (tracks 1–3), 5hmC% (track 4) from 6-base sequencing, gene expression (RNA-seq, tracks 5 and 6) with CpGs in *Foxo1* locus. 6-base sequencing; WT: naïve CD4^+^ T cells [CD4^+^ YFP(FOXP3)^−^ CD62L^high^ CD44^low^] from *Foxp3Cre WT* mice, DKO: Tfh like cells [CD4^+^ YFP(FOXP3)^−^ PD-1^+^ CXCR5^+^] from DKO-severe *Foxp3-Cre Tet2/3^fl/fl^* mice. RNA seq; WT: CD4^+^ YFP(FOXP3)^−^ T cells from *Foxp3Cre WT* mice, DKO: CD4^+^ YFP(FOXP3)^−^ T cells from DKO-severe *Foxp3-Cre Tet2/3^fl/fl^* mice. ChIP-seq; conventional CD4^+^ T cells were isolated from WT mice, and ChIP-seq for CTCF was performed (54).

TET enzymes deposit 5hmC at enhancers to maintain them in an active, demethylated state (43, 44). We examined DNA cytosine modification patterns in naïve CD4^+^ T cells (CD4^+^ YFP(FOXP3)^−^ CD62L^high^ CD44^low^) of 14-week-old WT mice and *Tet2/3 DKO* Tfh-like ex-Treg (CD4^+^ YFP(FOXP3)^−^ PD-1^+^ CXCR5^+^) cells of *Tet2/3 DKO*-severe mice using 6-base sequencing, a method that provides information on the four canonical bases—A, C, G, and T—and the modified bases 5mC and 5hmC in a single sequencing run (45) (**Figure 6**; **Supplementary Figure 6**). The results revealed several interesting and diverse ways in which methylation changes correlate with, and presumably modulate, changes in gene expression.

BCL6 is well-established as a lineage-defining transcription factor for Tfh cells (46–52). We observed a significant increase in *Bcl6* mRNA expression in Tfh-like ex-Treg cells in DKO-severe compared to naïve CD4^+^ cells (termed WT) (**Supplementary Figure 1D**; **Figures 2A**, **3C**), associated with a striking, unexpected *increase* in methylation of a region in the first intron of the *Bcl6* gene (**Figure 6A**, 5mC *tracks1–3* and RNA-seq, *tracks 4* and *5*). No significant changes were observed at the *Bcl6* promoter, which already lies within a wide “canyon” (53) of DNA demethylation in WT naïve CD4^+^ T cells (**Figure 6A**, 5mC, *track1*). Two previous studies may explain why the unexpected increase in 5mC correlated with increased gene expression: CTCF binds to the same first intron region of the *Bcl6* gene by CTCF chromatin immunoprecipitation (ChIP)-seq (54), and in a human B-cell lymphoma cell line, decreased CTCF binding correlated with hypermethylation of this region and increased *Bcl6* expression (55). Mouse CD4^+^ T cells and Treg cells do not express BCL6 and show high CTCF binding within and outside this intronic region (54) (shown for mouse CD4^+^ T cells in **Figure 6A**, ChIP-seq *tracks 6* and *7*). 5hmC levels in gene bodies (transcribed regions of genes), which correlate strongly with gene expression (56), are not shown for *Bcl6*: 5hmC is low to undetectable in naïve T cells (“WT” in **Figure 6A**), which do not express BCL6, as well as in *Tet2/3 DKO* cells, which have very low TET activity.

We also examined 5mC/5hmC distribution in the *Foxo1* gene, a well-known suppressor of Tfh differentiation in mice (48, 52). *Foxo1* mRNA is highly expressed in WT naïve CD4^+^ T cells, but downregulated in FOXP3^−^ ex-Treg cells from DKO-severe mice (**Figure 6B**, RNA-*seq tracks 5* and *6*)—consistent with the upregulation of Tfh-related genes observed in bulk and scRNA-seq (**Supplementary Figure 1D**; **Figure 3**). High *Foxo1* expression in WT T cells correlated with high 5hmC and relatively low 5mC in the gene body of *Foxo1* (**Figure 6B**, *5mC tracks 1–3* and *5hmC track 4*); the low to undetecTable 5hmC levels in *Foxp3-Cre Tet2/3 DKO* T cells are not shown. The downregulation of *Foxo1* mRNA expression in FOXP3^−^ *Tet2/3 DKO* ex-Treg cells correlated with increased methylation at the edges of the promoter “canyon” containing the 3′ of transcription start site (TSS) in the *Foxo1* gene (**Figure 6B**; 5mC *tracks1–3*), as previously noted for *Tet*-deficient cells. Thus, unlike the findings for *Bcl6*, increased methylation 3′ of the *Foxo1* TSS in *Tet2/3 DKO* T cells follows the expected paradigm, in which decreased gene expression correlates with increased DNA methylation within or at the edges of regulatory regions and in the transcribed body of the gene.

TOX2, MAF, and BATF are all transcription factors known to promote Tfh differentiation (47, 48, 57, 58), and the expression of all these transcription factors was strongly upregulated in DKO-severe compared to WT mice (**Figure 2A**; **Supplementary Figures 1D**, **6**, compare RNA-seq tracks). However, we again did not observe a simple correlation between changes in expression and changes in DNA methylation (5mC) in these genes. While the *Tox2* TSS was largely demethylated in both WT and *Tet2/3 DKO* cells, we observed increased methylation near the TSS and across the *Tox2* gene in *Tet2/3 DKO* compared to WT cells, especially at putative regulatory regions in the first, second, and third introns (**Supplementary Figure 6A**); this again contradicts the simple hypothesis that increased methylation, especially near putative enhancers or the TSS, is related to decreased gene expression and suggests an indirect effect. In contrast, there was no methylation at the 5′ UTR of the *Maf* gene in either WT or DKO cells, but decreased methylation in the gene body; the annotation of the *Maf* gene may be incorrect since transcription appears to start well 5′ of the annotated 5′ UTR (**Supplementary Figure 6B**). In the case of *Batf*, we observed both increased and decreased methylation at different regions, although RNA expression was unambiguously increased (**Supplementary Figure 6C**). These complex changes are discussed below.

## Discussion

We previously showed that TET-deficient Treg cells lose FOXP3 expression due to loss of TET activity, increased methylation of the conserved intronic *CNS1* and *CNS2* enhancers, and decreased FOXP3 stability (28), leading to the development of autoimmune-like symptoms in *Foxp3-CreTet2/3^fl/fl^* mice (28, 33). Although the onset was variable, the disease was fully penetrant and fatal within 6 months (33). In this study, we defined DKO-moderate and DKO-severe *Foxp3-CreTet2/3^fl/fl^* mice based on the total numbers of lymphocytes in the spleen at 14 weeks; these mice exemplify the early and late stages of disease progression, respectively. By comparing the phenotypes of DKO-moderate and DKO-severe mice, we found that FOXP3^−^ ex-Treg cells from *Foxp3-Cre Tet2/3 DKO* mice displayed progressive, time-dependent increases in multi-organ inflammation, based on substantial expansion of T cells with an activated phenotype and immunohistochemistry and histology showing splenomegaly, lymphadenopathy, and increased lymphocyte infiltration into kidneys, lungs, and liver. The variable yet progressive phenotype of *Tet2/3 DKO* mice resembles that of human patients with complex autoimmune/inflammatory disorders such as systemic lupus erythematosus (SLE), in whom autoimmune symptoms can manifest variably and may improve and worsen through multiple cycles of remission and relapse.

The slow increase in inflammation observed in DKO-moderate and DKO-severe *Foxp3-CreTet2/3^fl/fl^* mice is explained by our previous finding that Treg cells from these mice lose suppressive activity in a time-dependent manner compared to WT cells. This is apparent, for instance, from the decreased ability of *Tet2/3 DKO* (compared to WT) bone marrow cells to rescue the rampant inflammation and fatal inflammatory phenotype of newborn *scurfy* mice (33). Additionally, transfer of *Tet2/3 DKO* CD4^+^ T cells, including Treg cells and ex-Treg cells, into immunocompetent mice with a normal repertoire of functional Treg cells led to increased activation and expansion of bystander T cells in the recipient mice, indicating that *Tet2/3 DKO* cells are capable of conferring a dominant inflammatory phenotype even in immunocompetent mice with normal Treg function (33).

Notably, DKO-severe *Foxp3-CreTet2/3^fl/fl^* mice showed significantly increased frequencies and absolute numbers of CD4^+^ T cells expressing Tfh markers (PD-1, CXCR5, and ICOS), as judged using flow cytometry and immunohistochemistry. CD4^+^ T cells from these mice (which include FOXP3^−^ “ex-Treg” cells as well as conventional activated CD4^+^ T cells) displayed increased IL-21 transcription and secretion and increased expression of Tfh-associated genes compared to CD4^+^ FOXP3^−^ cells of WT mice based on flow cytometry of stimulated cells and both bulk and single-cell RNA-seq. The mice also displayed a progressive increase in total numbers and frequencies of plasma cells in the secondary lymphoid tissue and peripheral tissues, which was especially apparent in DKO-severe mice. Furthermore, mice with the DKO-severe phenotype showed a convincing increase in circulating autoantibodies that was variable with respect to the exact autoreactivity detected. Tfh cells provide help to germinal center B cells, which then differentiate into plasma cells, and plasma cells residing in inflamed tissues produce antibodies in chronic inflammatory and systemic autoimmune diseases. Increased autoantibody production was indeed observed in *Tet2/3 DKO* mice, especially those categorized as DKO-severe. The parallel increase in cytotoxic CD8^+^ T cells expressing high levels of perforin and granzyme B could be due to the emergence of Tfh-like cells that produce IL-21, which has been shown to induce cytotoxic effector function in CD8^+^ T cells via the upregulation of key molecules such as IFN-γ and granzyme B in anti-tumor immunity or viral infection (59, 60).

In contrast, the frequency of CD4^+^ cells expressing IL-17 after stimulation was very low in DKO-severe *Foxp3-CreTet2/3^fl/fl^* mice, and the frequency of IFNγ^+^ cells after stimulation declined as the disease progressed. Bulk RNA-seq suggested that only one of four mice examined expressed significant baseline levels of IL-17 or *Rorc*, and very few *Rorc*-expressing cells were detected in CD4^+^ FOXP3^−^*Tet2/3 DKO* T cells via scRNA-seq using the sequence barcode attached to each cell, which allowed us to distinguish WT and *DKO* cells (range 0.2% to 3% *Rorc^+^* cells). Since the CD4^+^ cells in these mice include FOXP3^−^ “ex-Treg” cells as well as conventional activated CD4^+^ T cells, the variability may reflect differences in the relative proportions of these two cell types. Analysis of cluster 3, a cluster of Tfh-like cells mostly from DKO ex-Treg cells, showed that only 1.5% of *DKO* cells expressed *Rorc*. Together, the data suggest that *Rorc* is not as highly expressed as Tfh markers and poorly expressed in Tfh-like ex-Treg cells. This finding is consistent with previous reports that Tfh-related transcription factors such as BCL6 and TOX2 suppress *Rorc* expression and Th17 differentiation (48, 57, 61, 62).

How does the biochemical activity of TET proteins contribute to autoimmune disease? The answer may lie in the genes in any given cell type that are most sensitive to loss of TET activity. The primary biochemical effect of TET proteins is to maintain low levels of DNA methylation at gene promoters, transcribed regions (gene bodies), enhancers, and other regulatory regions. Changes in gene expression were clearly associated with aberrant (often subtle) changes in DNA methylation in and near gene bodies—increased 5mC at the edges of demethylated regions surrounding transcription start sites or putative intragenic enhancers. Some of these demethylated regions fit the definition of “canyons”, which in other systems are observed near genes strongly involved in cell lineage specification (53). Notably, however, there was not always a clear connection between changes in DNA methylation and gene expression. To illustrate, we observed a striking accumulation of 5mC in the first intron of the *Bcl6* gene, associated with increased expression of *Bcl6* mRNA (**Figure 6A**). This finding does not support the paradigm that increased DNA methylation is always suppressive for gene expression. Our data support the hypothesis that hypermethylation interferes with CTCF binding to an intronic region of the *Bcl6* gene in both humans and mice (54, 55, 63, 64). CD4^+^ T cells from older (middle-aged) Tet2^gt^ gene trap hypomorphic mice, which had 20%–30% residual *Tet2* gene expression compared to WT T cells, showed significant Tfh cell expansion with hypermethylation of the same intronic region of the *Bcl6* gene (63, 64). Likewise, Raji human B-cell lymphoma cells showed increased BCL6 expression, increased intronic hypermethylation, and decreased CTCF binding relative to H929 plasmacytoma cells, which express lower levels of BCL6 (55). Potentially, CTCF binding to the unmethylated intronic regions forms a barrier between a distal enhancer (or a TAD boundary) and the gene promoter, as shown for imprinted genes and for certain oncogenes (65); hypermethylation results in decreased CTCF binding and increased gene expression.

In contrast, decreased *FOXO1* expression in *Foxp3-Cre Tet2/3 DKO* CD4^+^ T cells showed the “expected” correlation with increased methylation across the transcribed region of the *FOXO1* gene. Moreover, the almost complete loss of FOXO1 in DKO-severe mice correlated with the almost total loss of 5hmC across the genome of *Foxp3-Cre Tet2/3 DKO* mice; the presence of 5hmC is one of the best markers for genes that are actively being transcribed. FOXO1 and the RUNX1-related transcription factors RUNX2 and RUNX3 are well-known suppressors of Tfh differentiation in mice (48, 52): FOXO1 coordinates with TET2 to mediate the demethylation and expression of the *Runx2* and *Runx3* genes (66). *Tet2*-deficient CD4^+^ T cells from SMARTA TCR transgenic mice (specific for an lymphocytic choriomeningitis virus (LCMV) GP66–77 epitope) showed preferential differentiation into Tfh cells upon LCMV infection, rather than predominantly into Th1 cells as in virally infected wild-type mice (66, 67); this correlated with a failure to demethylate the *Runx2* and *Runx3* promoters and decreased expression of *Runx2* and *Runx3* mRNA (66). In our own studies, DNA methylation at the *Runx2/3* promoters was increased, but *Runx2/Runx3* mRNA expression was not changed in DKO-severe CD4^+^ FOXP3-negative ex-Treg cells compared to WT cells (data not shown). FOXO1 could act alone without a requirement for RUNX2 and RUNX3; alternatively, because FOXO1 is critical for T-cell quiescence (68), decreased FOXO1 expression in normally quiescent Treg cells would contribute to their proliferation in *Foxp3-Cre Tet2/3 DKO* mice, and concomitant increased expression of BCL6 and other effectors of Tfh lineage programs would result in Tfh skewing.

Expansion of the Tfh population was observed in both FOXP3^+^ Treg cells that had not yet lost FOXP3, as well as FOXP3^−^ cells in DKO-severe mice. We infer that the process of Tfh differentiation was initiated in FOXP3^+^ Treg cells before FOXP3 loss was complete. We cannot rule out, however, that conventional CD4^+^ T cells also undergo Tfh skewing—*Tet2 KO* naïve CD4^+^ T cells display a cell-intrinsic bias toward Tfh differentiation upon LCMV infection, with impaired Th1 differentiation (66, 67), and TET deficiency suppresses Th1, Th2, and Th17 differentiation from naïve cells *in vitro* (69). Leaky expression of *Foxp3-Cre*, which occurs at low stochastic levels during hematopoietic development (37, 38), may also be responsible for some aberrant lineage specification in *Foxp3-Cre Tet2/3^fl/fl^* mice. To counter this problem, we are implementing a new strategy for ex-Treg development in which purified Treg cells from *CD4-Cre Tet2/3^fl/fl^* mice (not leaky for TET deletion in other lineages) are adoptively transferred into new recipient mice (K. Suzuki, *unpubl.*).

We previously reported that expansion of iNKT cells in *CD4-CreTet2/3^fl/fl^* mice required antigenic stimulation since it did not occur in mice lacking the non-classical MHC protein CD1d, which presents lipid antigens to iNKT cells (34, 70). Further, we have observed that bona fide resident Tfh cells show striking expansion in *CD4-CreTet2/3^fl/fl^* mice. *Tet2/3 DKO* ex-Treg cells display increased clonal expansion (33); in fact, proliferation of specific cell types in response to signals encountered *in vivo* is a recurrent feature of TET deficiency in mouse models (34, 70, 71), and TET2 loss-of-function mutations are frequently observed in human hematopoietic cancers (43, 72). Since TET proteins play important roles in thymic Treg differentiation, TET deficiency may be implicated in the emergence of self-reactive CD4^+^ FOXP3^+^ T cells in *Foxp3-Cre Tet2/3^fl/fl^* mice. Thus, self-reactive *Tet2/3 DKO* Tregs in the thymus may expand and generate Tfh-like ex-Treg cell clones in the periphery, and this may lead to expansion of self-reactive Tfh cells and autoantibody-producing plasma cells in *Foxp3-Cre Tet2/3 DKO* mice. The random emergence of different self-reactive clones in individual mice could contribute to the variable autoimmune phenotypes of *Foxp3-Cre Tet2/3 DKO* mice.

In summary, the mechanisms by which increased DNA methylation due to TET loss-of-function results in changes in gene expression, lineage skewing, and inflammation are still unclear. Gene expression is strongly influenced by changes in the binding of transcriptional regulators to distal, proximal, or intragenic enhancers/regulatory regions that are specific to each gene. Hence, to understand the influence of DNA methylation changes on gene regulation and lineage specification in depth, it will be necessary to identify transcriptional regulators that govern the expression of each differentially expressed gene and to dissect how DNA methylation at promoters, enhancers, and gene bodies influences the binding of these regulators to gene promoters and enhancers. In humans, changes in enhancer activity can be traced to single-nucleotide polymorphisms in putative enhancers identified by Genome-Wide Association Study (GWAS), suggesting that it would be worthwhile to investigate changes in DNMT/TET activity and regulation, as well as changes in DNA methylation in human autoimmune disease. This is especially interesting since the C>T transitions that are frequent in GWAS are thought to reflect deamination of methylated CA and CpG sequences during evolution (73, 74).

Recently, *TET2* has been identified as the second most frequent mutation (after *DNMT3A*) in age-related clonal hematopoiesis [ARCH or CH, also known as clonal hematopoiesis of indeterminate potential (CHIP)] (75). In CH, somatic *TET2* mutations cause hematopoietic stem cell (HSC) clones to expand. *TET2*-deficient HSCs exhibit enhanced self-renewal capability and undergo myeloid skewing with clonal expansion. In addition, chronic inflammation is promoted by *TET2*-deficient myeloid cells through the production of inflammatory cytokines such as IL-1β, and it has been shown that individuals with CHIP have an increased risk of atherosclerosis and cardiovascular disease (76, 77). Indeed, patients with SLE and RA show a higher prevalence of CHIP compared with healthy controls of the same age (78, 79). Particularly in rheumatoid arthritis (RA), a subgroup with late-onset disease has been observed, and patients over 60 years old develop the disease. Age-associated CH/CHIP driven by TET2 or DNMT3A deficiency may contribute to the development of late-onset autoimmune disease.

## Data Availability

The data presented in the study are deposited in the NCBI GEO repository, accession numbers GSE325544, GSE325568 and GSE325631.

## Supplementary material

Supplementary Figure 1 **(A)**. Survival curves for control WT (n=28) and *Foxp3-Cre Tet2/3^fl/fl^* mice separated into female and male groups (n=14 for each group) reproduced from (33). **(B–D)**. RNA-seq analysis for CD4^+^ YFP(FOXP3^-^) T cells from *Foxp3Cre WT* mice and *Foxp3-Cre Tet2/3^fl/fl^* mice (14-weeks-old). **(B)** PCA plot. **(C, D)** Mean average (MA) plot of genes differentially expressed in DKO-moderate relative to their expression in WT **(C)**, DKO-severe relative to their expression in WT **(D)**.

Supplementary Figure 2 **(A-C)** Flow cytometry analysis of cytokines in CD4^+^ and CD8^+^ cells. IL-17A^+^ cells and IFN-γ^+^ cells with PMA and ionomycin stimulation (PMA iono+) (gated on TCRβ^+^ CD4^+^ cells) **(A)**, IL-17A^+^ and IFN-γ^+^ cells with PMA and ionomycin stimulation (gated on TCRβ^+^ CD8^+^ cells) **(B)**, Perforin^+^ and Granzyme B^+^ cells without PMA and ionomycin stimulation (PMA iono-) (gated on TCRβ^+^ CD8^+^ cells) **(C)** in pooled spleen and peripheral lymph nodes (cervical and inguinal) of from 14-weeks-old *Foxp3Cre WT* and *Foxp3-Cre Tet2/3^fl/fl^* mice. **(D, E)** Quantification of the frequency of IL-17A^+^ cells **(D)**, IFN-γ^+^ cells **(E)** in CD4^+^ T cells. **(F–H)** IFN-γ^+^ **(F)**, Perforin^+^ cells **(G)**, Granzyme B^+^ cells **(H)** in CD8^+^ T cells. **(I)** Flow cytometry analysis of cytokines in CD4^+^ cells. IL-21^+^ cells with or without PMA and ionomycin stimulation (PMA iono+/-) (gated on TCRβ^+^ CD4^+^ cells) **(J)** Quantification of the frequency of B220^+^ cells in live cells and absolute number. **(K)** Flow cytometry analysis of GC-B cells (Fas^+^ GL-7^+^ cells) (gated on B220^+^ cells) in pooled spleen and peripheral LNs (cervical and inguinal) from 14-weeks-old *Foxp3Cre WT* and *Foxp3-Cre Tet2/3^fl/fl^* mice (*left*). Quantification of the frequency of Fas^+^ GL-7^+^ cells in B220^+^ T cells.

Supplementary Figure 3 **(A, B)** The graphs show the *Tet2* expression in *Tet2/3 DKO* Treg cells relative to WT Treg cells **(A)**, the *Tet2* expression in *Tet2/3 DKO* non-Treg cells relative to WT non Treg cells **(B)**. Treg cells: CD4^+^ YFP(FOXP3)^+^, WT non Treg cells: CD4^+^ YFP(FOXP3)^-^, Tfh cells: CD4^+^ YFP(FOXP3)^-^ PD-1^+^ CXCR5^+^, non Tfh cells: CD4^+^ YFP(FOXP3)^-^ PD-1^-^ CXCR5^-^, Naïve CD4^+^ cells: CD4^+^ YFP(FOXP3)^-^ CD62L^high^ CD44^low^. **(C)** Data of *Tet2* or *Tet3* expression in each cell type from DICE (Database of Immune Cell Expression, Expression quantitative trait loci (eQTLs) and Epigenomics) project. **(D)** Representative flow cytometry plots of PD-1^+^ CXCR5^+^ cells (gated on TCRβ^+^ CD4^+^ cells) and quantification of the frequency of PD-1^+^ CXCR5^+^ cells in pooled spleen and peripheral lymph nodes (cervical and inguinal) from 4–6 weeks old *Tet2/3^fl/fl^* (WT) and *Cd4-Cre Tet2/3^fl/fl^* mice. **(E)** Representative flow cytometry plots of Rosa26-YFP^+^ cells (gated on PD-1^+^ CXCR5^+^ in CD4^+^ FOXP3^-^ T cells) and quantification of the frequency of the cells in pooled spleen and peripheral lymph nodes (cervical and inguinal) from 14 weeks old Heterozygous (*Foxp3-Cre Tet2/3^fl/+^ Rosa26-YFP^LSL^*), DKO-moderate or DKO-severe *Foxp3-Cre Tet2/3^fl/fl^ Rosa26-YFP^LSL^* mice.

Supplementary Figure 4 Heatmap from polyreactivity assay for serum isolated from WT (n=8) and DKO-moderate (n=3), DKO-severe (n=4) tested by autoantibody array assay kit (13–15 weeks old mice). For data analysis, R package ‘limma’ and multiple comparisons correction was performed (adjusted p value < 0.05 between tested groups).

Supplementary Figure 5 H&E staining of Kidney, Liver, Lung and Pancreas from 14-week-old *Foxp3Cre WT* and *Foxp3-Cre Tet2/3^fl/fl^* mice. Scale bar; Kidney: 100μm, Liver: 200μm, Lung: 250μm, Pancreas: 50μm.

Supplementary Figure 6 **(A–C)** Genome browser views showing 5mC% (track 1-3) from 6 base sequencing, gene expression (RNA-seq, track 4-5) in *Tox2* **(A)**, *Maf* **(B)** and *Batf* **(C)** locus. 6 base sequencing; WT: naïve CD4^+^ T cells (CD4^+^ YFP(FOXP3)^-^ CD62L^high^ CD44^low^) from *Foxp3Cre WT* mice, DKO: Tfh like cells (CD4^+^ YFP(FOXP3)^-^ PD-1^+^ CXCR5^+^) from DKO-severe *Foxp3-Cre Tet2/3^fl/fl^* mice. RNA seq; WT: CD4^+^ YFP(FOXP3)^-^ T cells from *Foxp3Cre WT* mice, DKO: CD4^+^ YFP(FOXP3)^-^ T cells from DKO-severe *Foxp3-Cre Tet2/3^fl/fl^* mice.
