# Supplementary material for: Loss of TET function in T regulatory cells yields ex-Treg cells biased toward T follicular helper cells, causing autoimmune diseases through autoantibody production
Source: Front Immunol. 2026 Mar 27;17:1684023. doi: 10.3389/fimmu.2026.1684023 (PMC13066302; doi:10.3389/fimmu.2026.1684023)
Supplement: Supplementary file 1 [file Supplementaryfile1.docx]

**Material and Method**

**Mice.**

B6.129(Cg)-*Foxp3tm4(YFP/icre)Ayr/J* (Foxp3-Cre, strain 016959) were obtained from Jackson Laboratory. *Foxp3-Cre Tet2^fl/fl^ Tet3^fl/fl^* mice were generated in our laboratory by crossing *Tet2^fl/fl^ Tet3^fl/fl^* with *Foxp3-Cre* mice. For the fate-mapping experiments, Tet2fl/fl Tet3+/fl Foxp3Cre female mice were crossed with Tet2fl/fl Tet3fl/fl Rosa26-YFP+ male mice to generate Tet2fl/fl Tet3fl/fl Foxp3Cre Rosa26-YFP+ male mice for further analysis. All mice were on the B6 background and maintained in a specific pathogen-free animal facility in the La Jolla Institute for Immunology. Age of the mice used for each experiment was stated in the figure legends. All animal procedures were reviewed and approved by the Institutional Animal Care and Use Committee of the La Jolla Institute for Immunology and were conducted in accordance with institutional guidelines. Euthanasia methods complied with the AVMA Guidelines for the Euthanasia of Animals. Euthanasia was induced by carbon dioxide (CO_2_) asphyxiation/anesthetic overdose coupled with cervical dislocation. The carbon dioxide displacement rate was from 30% - 70% of the chamber volume per minute. For the euthanasia by anesthetic overdose, 5% isoflurane delivered by precision vaporizer. Cervical dislocation was performed as a secondary step to ensure euthanasia.

**Cell preparation and flow cytometry.**

Single-cell suspensions were prepared from pooled spleen and peripheral lymph nodes (cervical and inguinal) for staining or cell sorting. For analysis of T cell compartments and Treg cell features, single-cell suspensions were stained with anti-mouse antibodies against the following (Clone name, conjugated fluorescence, dilution, manufacturer and catalog number shown in brackets): CD4 (GK1.5, BV421, 1:200, Biolegend, #100443; RM4-5, BV605, 1:200, Biolegend, #100548), CD8 (53-6.7, PE-Cy7, 1:200, Biolegend,#100722), TCRβ (H57-597, BV421, 1:200, Biolegend, #109230; H57-597, PE, 1:200, Biolegend, #109208), CD62L (MEL-14, APC, 1:200, Biolegend, #104412), CD44 (PerCP-Cy5.5, 1:200, Biolegend, #103032), B220 (RA3-6B2, APC, 1:200, Biolegend, #103212), Gr1 (RB6-8C5, BV650, 1:200, Biolegend, #108442), CD11b (M1/70, PerCP-Cy5.5, 1:200, Biolegend, #101228), CD98 (RL388, PE-Cy7, 1:200, Biolegend, #128213), CD138 (281-2, BV421, 1:200, Biolegend, #142507), PD-1 (29F.1A12, PE-Cy7, 1:200, Biolegend, #135216; J43, APC, 1:200, Invitrogen, 17-9985-80), CXCR5 (SPRCL5, Biotin, 1:200, Invitrogen, #13-7185-82), ICOS (C398.4A, BV785, 1:200, Biolegend, #313533), BV421 Streptavidin (1:200, Biolegend, #405225) antibodies. For intracellular staining, cells were surface-stained and then stained with anti-BCL6 (IG191E/A8, PE, 1:100, Biolegend, #648303), anti-IL-21 (mhalx21, PE, 1:100, Invitrogen, # 12-7213-82), anti-IL17A (TC11-18H10.1, PerCP-Cy5.5, 1:100, Biolegend, #506920), anti-IFN-γ (TC11-18H10.1, APC, 1:100, Biolegend, #505810), anti-Perforin (S16009A, PE/Dazzle594, 1:100, Biolegend, #154315), anti-Granzyme B (QA18A28, Pacific Blue, 1:100, Biolegend, #396420), antibodies. The cell were fixed 4% PFA and permed permeabilization buffer (50mM NaCl, 5mM EDTA, 0.02% NaN3, 0.5% TritonX) and analyzed by flow cytometry on Celesta, LSR-II and Fortessa from BD.

**RNA-sequencing library preparation.**

Total RNA was isolated from CD4^+^ YFP(FOXP3^-^) T cells from pooled spleen and peripheral lymph nodes (cervical and inguinal) from *Foxp3Cre WT* mice and *Foxp3-Cre Tet2/3^fl/fl^* mice (14-weeks-old) using Direct-zol RNA microprep (Zymo, #R2060). RNA-sequencing libraries were prepared using NEBNext Ultra II Directional RNA Library Prep Kit for Illumina (New England Biolabs, #E7760, #7765) according to the manufacture’s protocol and sequenced at the La Jolla Institute sequencing core using Illumina NovaSeq 6000 pair end 150 bp platform.

**RNA-sequencing analysis.**

Reads were aligned to the mouse reference genome mm10 using STAR version 2.7.11 (1) with the parameters -outFilterMultimapNmax 1 -alignIntronMin 20 -twopassMode Basic -outBAMcompression 10 -alignSJoverhangMin 8 -alignSJDBoverhangMin 1 -outFilterType BySJout -outSJfilterReads Unique -outFilterMismatchNoverReadLmax 0.04 to analyze gene expression. HT-Seq (2) was used to quantify the gene expression levels using the options htseq-count --stranded reverse. Genes with at least ten read counts in one condition were kept for further analysis. Normalization and differential expression analyses were performed using DESEq2 (3). For visualization of the data, we generated tracks using Deeptools94 version 3.5.1, with the option bamCoverage. All related plots were made using R-Studio95 and Integrative Genome Viewer96 version 2.16.0.

**Single-cell RNA-sequencing library preparation and analysis.**

TCRβ^+^ cells from pooled spleen and peripheral lymph nodes (cervical and inguinal) of *Foxp3Cre WT* and DKO-severe *Foxp3-Cre Tet2/3^fl/fl^* mice (14-weeks-old) were prepared, counted and loaded 10,000 single cells (n=1). Samples were processed per the manufacturer’s protocol using Chromium Next GEM Single Cell 3’ Kit v3.1 (10X Genimics) and sequenced were conducted at the La Jolla Institute sequencing core on an Illumina Novaseq 6000 sequencer using a 100-bp kit. Samples were sequenced using a 28|10|10|90 run configuration. Sample demultiplexing, barcode processing, alignment, filtering, UMI counting and aggregation of sequencing runs were performed using the Cell Ranger analysis pipeline (v.7.1.0) and the mouse reference genome mm10. Downstream analysis was performed using the R package Seurat (4).

**6-base sequencing (simultaneous detection of 5hmC and 5mC by duet multiomics solution evoC (biomodal)) library preparation and analysis.**

Naïve CD4^+^ T cells from *Foxp3Cre WT* mice (14-weeks-old) and Tfh cells from DKO-severe *Foxp3-Cre Tet2/3^fl/fl^* mice (14-weeks-old) were sorted and extracted genomic DNA using DNeasy Blood & Tissue Kit (Qiagen, #69504). The duet multiomics solution evoC method (6-base sequencing) was performed according to the manufacturer’s instructions (duet exoC User guide v.3), using 60-70 ng of sonicated genomic DNA as starting material (n=2). Libraries were sequenced at the UCSD Institute for Genomic Medicine Genomics Center using Illumina NovaSeq X Plus pair end 150 bp platform. Data were processed following the duet evoC analysis pipeline v1.4.1. Downstream analysis of the data was performed as in ref. (5) and the mean coverage for the samples ranged from 10x to 20x.

**Histology.**

Pancreas, colon, skeletal muscle, heart, liver, lung, salivary gland, brain, knee joint, stomach were isolated from 14-week-old *Foxp3Cre WT* mice and *Foxp3-Cre Tet2/3^fl/fl^* mice. Samples were fixed using 10% zinc formalin, washed in 70% isopropanol, processed and embedded in paraffin. The blocks were then cut and 4 µm sections were stained with haematoxylin and eosin (H&E) (6). Slides were digitized with ZEISS AxioScan.Z1 scanner equipped with a 20x 0.8NA objective. Whole slide images were analyzed by a board-certified veterinary pathologist in QuPath (7) software. Neoplastic changes were not seen and inflammation was scored on a 0-5 scale for quantity of infiltrating cells including neutrophils, lymphocytes, macrophages, and plasma cells in specified regions of the analyzed tissues.

**Orion multiplexing.**

Five-micron sections were placed on positively charged slides, dried, and baked at 60°C for 1h to adhere tissue, then deparaffinized using three cycles of Pro-Par clearant dipping (20×) and submersion (10min). Slides underwent rehydration in reagent alcohol: twice in 100% (20× dips, 1.5min submersion) and once in 90% (20× dips, 1.5min submersion), followed by a 2min DI water rinse. Antigen retrieval was performed in pH 6 citrate buffer in a Biocare Medical Decloaking Chamber™ NxGen (Program 5: 110°C, 15min) and then cooled to RT. Autofluorescence was quenched in PBS with 2.4 mM NaOH and 1.47 M H_2_O_2_ solution under LED (1h) and UV light (30min). Slides were placed in a Freequenza rack (8), blocked with Image-iT FX Signal Enhancer (15min), and washed (1mL, 0.025% TritonX-100 in PBS). Slides were stained with a 16-plex conjugated antibody panel, including anti-mouse CD3ε (D4V8L)-ArgoFluor 548, CD8α (4SM15)-ArgoFluor 658, B220 (RA3-6B2)-ArgoFluor 698, IRF4 (E8H3S)-ArgoFluor 706, FOXP3 (D6O8R)-DIG-ArgoFluor 760, CD4 (4SM95)-ArgoFluor 812, CD31 (D8V9E)-ArgoFluor 874 and others in Candor Antibody Stabilizer PBS with 5% mouse and 5% rabbit serum overnight at 4°C. After warming to RT and washing (4mL), slides were stained with secondary antibodies in Antibody Stabilizer PBS with 10% goat serum (30min) and washed (4mL). Slides were counterstained with Hoechst (1:1000, 5min), washed (5mL PBS), coverslipped with ArgoFluor Mounting Medium, and dried for 48h before scanning. Images were acquired with a 20× 0.75 dry objective (0.325μm/pixel) using whole-tissue tiling. The Orion system (RareCyte, Seattle, Washington, USA) captured ~10nm emission bands via angled dichroic mirrors. Processing used Rarecyte's algorithm for tile stitching, non-linear channel alignment, and spectral unmixing based on previously acquired single-color controls. The Orion images were analyzed in QuPath 0.6.0 (9). For cell counting, images were analyzed in QuPath 0.6.0 (9). Briefly, cells were segmented using InstanSeg (10). Random forest machine learning classifiers were trained to identify cells positive for each of ICOS, CD3 (hi vs low), CD4, CD8, and IRF, as well as cells simultaneously negative for CD3ε, B220, and F4/80. Ex-Tregs were defined as ICOS^+^ CD3ε^+^ CD4^+^ FOXP3^-^ cells. Plasma cells were defined as IRF^+^ CD3ε^-^ B220^-^ F4/80^-^.

**Measuring auto-antibody level in serum.**

Blood was collected by cardiac puncture in BD Microtainer (BD, #365978) and serum was isolated. Autoantibodies level in serum was measured using Human Autoimmune Disease IgG Autoantibody array G1 (RayBiotech, #PAH-AIDG-G1-16) according to the manufacture’s protocol. We used biotin-conjugated Anti-mouse IgG (Jackson ImmunoResearch, #115-065-071) instead of biotin-conjugated Anti-human IgG to measure mouse auto-antibodies.

**Quantitative real-time PCR.**

Total RNA was isolated using Direct-zol RNA microprep (Zymo, #R2060); cDNA was synthesized using SuperScript™ IV First-Strand Synthesis System (Thermo Fisher, #18091050). Quantitative real-time PCR was performed using PowerUp™ SYBR™ Green Master Mix for qPCR (Thermo Fisher, #A25742) on a StepOnePlus real-time PCR machine (Applied Biosystems). Gene expression was normalized to *Gapdh*. Primers used to detect the expression levels of *Tet2* are as following:

Tet2 forward primer: AACCTGGCTACTGTCATTGCTCCA

Tet2 reverse primer: ATGTTCTGCTGGTCTCTGTGGGAA

*Gapdh* forward primer: TCACCACCATGGAGAAGGC

*Gapdh* reverse primer: GCTAAGCAGTTGGTGGTGCA

**Statistics.**

*P* values from one-way ANOVA test were used for all the statistical comparisons between different groups and data were displayed as mean ± SEM. (Prism). *P* values are denoted in corresponding figures as: **P* < 0.05, ***P* <0.01, ****P* < 0.001, *****P* < 0.0001.

**References (Material and Method):**

1. Dobin A, Davis CA, Schlesinger F, Drenkow J, Zaleski C, Jha S, et al. STAR: ultrafast universal RNA-seq aligner. Bioinformatics. 2013 Jan 1;29(1):15–21.

2. Anders S, Pyl PT, Huber W. HTSeq—a Python framework to work with high-throughput sequencing data. Bioinformatics. 2015 Jan 15;31(2):166–9.

3. Love MI, Huber W, Anders S. Moderated estimation of fold change and dispersion for RNA-seq data with DESeq2. Genome Biol. 2014 Dec 5;15(12):550.

4. Stuart T, Butler A, Hoffman P, Hafemeister C, Papalexi E, Mauck WM, et al. Comprehensive Integration of Single-Cell Data. Cell. 2019 June;177(7):1888-1902.e21.

5. Füllgrabe J, Gosal WS, Creed P, Liu S, Lumby CK, Morley DJ, et al. Simultaneous sequencing of genetic and epigenetic bases in DNA. Nat Biotechnol. 2023 Oct;41(10):1457–64.

6. Angela Denn. Hematoxylin & Eosin Protocol For Leica ST5020 Automated Stainer v2. 2023 Nov22. Available from dx.doi.org/10.17504/protocols.io.x54v9mozqg3e/v2

7. Bankhead P, Loughrey MB, Fernández JA, Dombrowski Y, McArt DG, Dunne PD, et al. QuPath: Open source software for digital pathology image analysis. Sci Rep. 2017 Dec 4;7(1):16878.

8. Meyer M, Dobaczewska K, Mikulski Z. Freequenza Box - Production and Assembly v1. 2022 Feb 17. Available from: https://www.protocols.io/view/freequenza-box-production-and-assembly-byqmpvu6

9. Bankhead P, Loughrey MB, Fernández JA, Dombrowski Y, McArt DG, Dunne PD, et al. QuPath: Open source software for digital pathology image analysis. Sci Rep. 2017 Dec 4;7(1):16878.

10. Goldsborough T, O’Callaghan A, Inglis F, Leplat L, Filby A, Bilen H, et al. A novel channel invariant architecture for the segmentation of cells and nuclei in multiplexed images using InstanSeg. bioRxiv 2024 Sep 8. Available from: http://biorxiv.org/lookup/doi/10.1101/2024.09.04.611150
