# Supplementary figures and images for: Loss of TET function in T regulatory cells yields ex-Treg cells biased toward T follicular helper cells, causing autoimmune diseases through autoantibody production
Source: Front Immunol. 2026 Mar 27;17:1684023. doi: 10.3389/fimmu.2026.1684023 (PMC13066302; doi:10.3389/fimmu.2026.1684023)

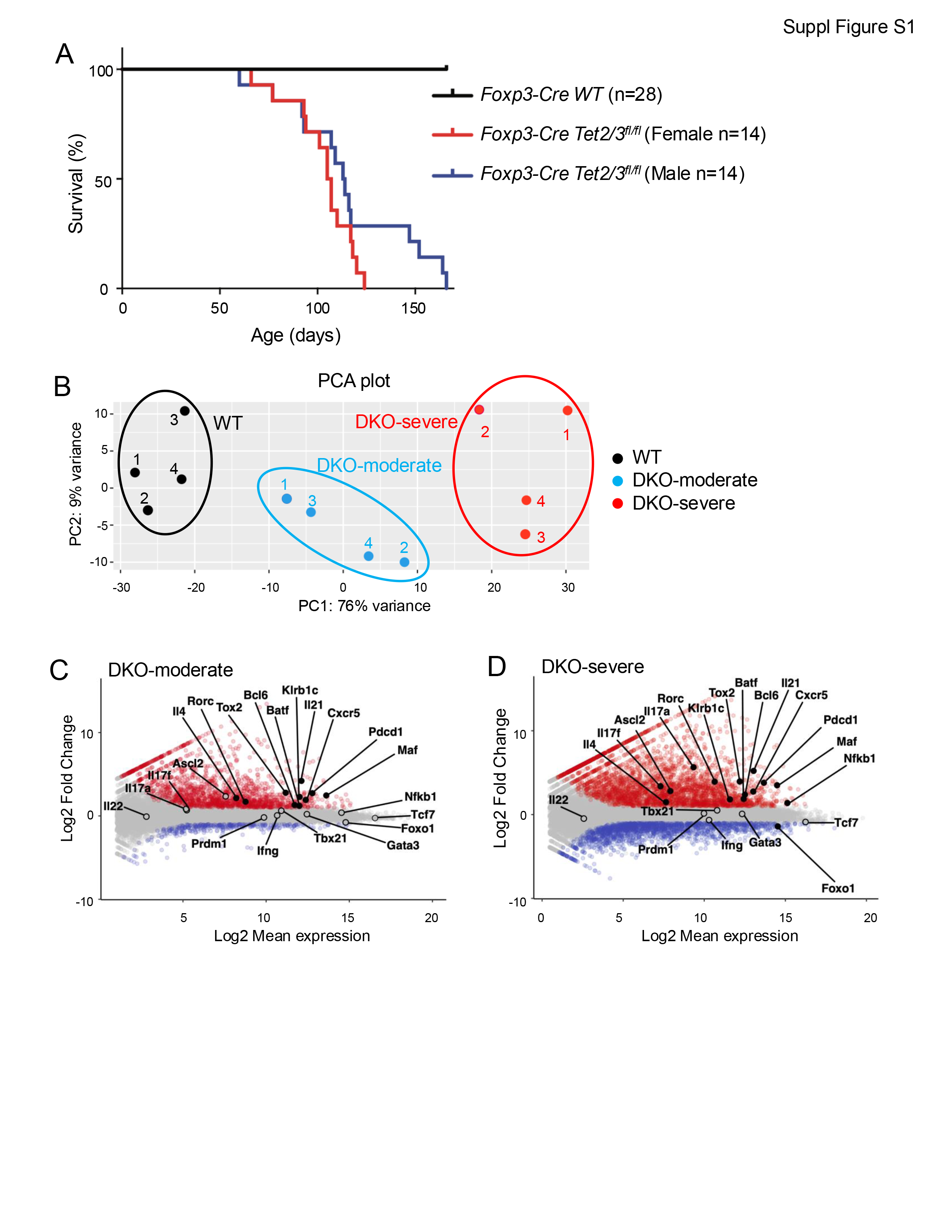

Supplement: Supplementary Figure 1 — (A). Survival curves for control WT (n=28) and Foxp3-Cre Tet2/3fl/fl mice separated into female and male groups (n=14 for each group) reproduced from (33). (B–D). RNA-seq analysis for CD4+ YFP(FOXP3-) T cells from Foxp3Cre WT mice and Foxp3-Cre Tet2/3fl/fl mice (14-weeks-old). (B) PCA plot. (C, D) Mean average (MA) plot of genes differentially expressed in DKO-moderate relative to their expression in WT (C), DKO-severe relative to their expression in WT (D). [file Image1.tif]

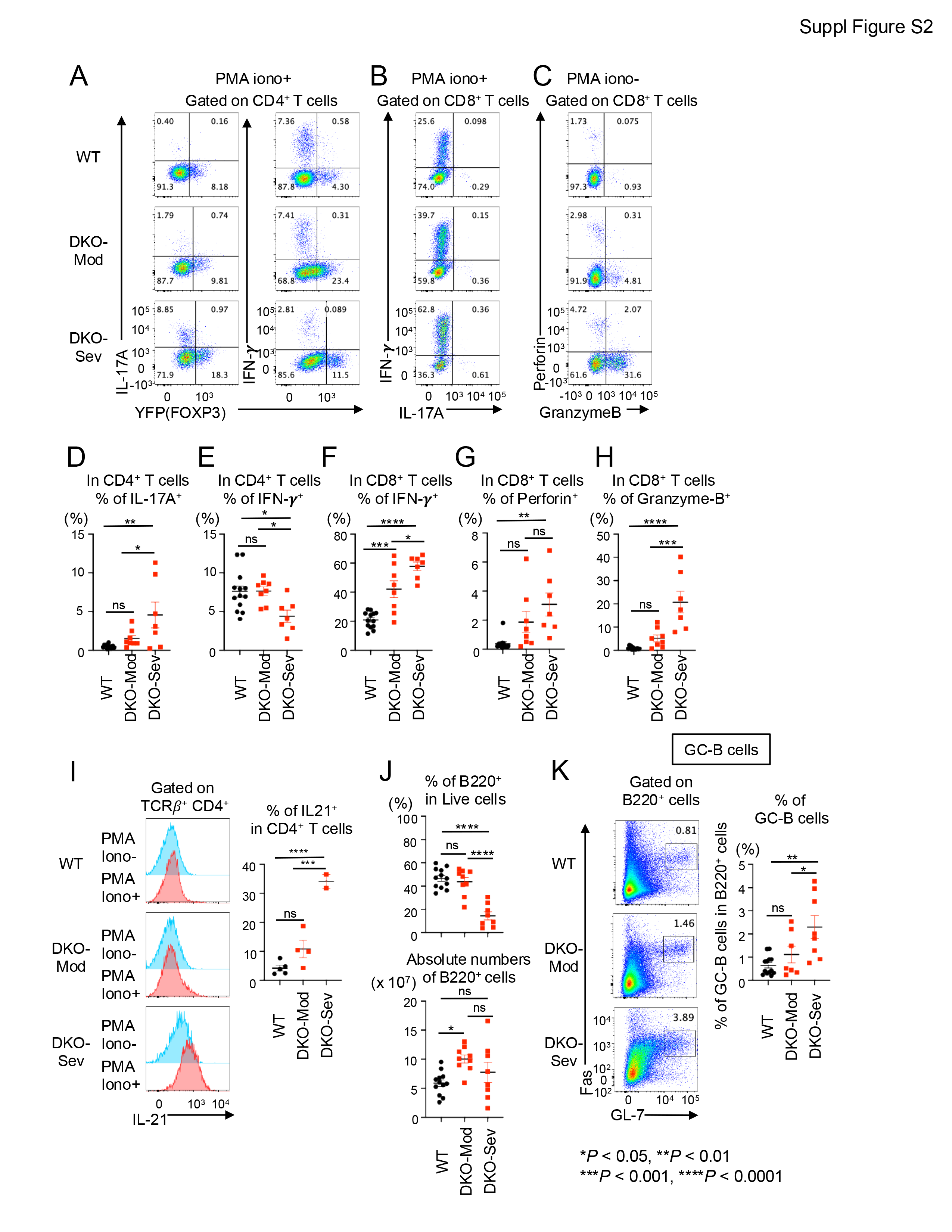

Supplement: Supplementary Figure 2 — (A-C) Flow cytometry analysis of cytokines in CD4+ and CD8+ cells. IL-17A+ cells and IFN-γ+ cells with PMA and ionomycin stimulation (PMA iono+) (gated on TCRβ+ CD4+ cells) (A), IL-17A+ and IFN-γ+ cells with PMA and ionomycin stimulation (gated on TCRβ+ CD8+ cells) (B), Perforin+ and Granzyme B+ cells without PMA and ionomycin stimulation (PMA iono-) (gated on TCRβ+ CD8+ cells) (C) in pooled spleen and peripheral lymph nodes (cervical and inguinal) of from 14-weeks-old Foxp3Cre WT and Foxp3-Cre Tet2/3fl/fl mice. (D, E) Quantification of the frequency of IL-17A+ cells (D), IFN-γ+ cells (E) in CD4+ T cells. (F–H) IFN-γ+ (F), Perforin+ cells (G), Granzyme B+ cells (H) in CD8+ T cells. (I) Flow cytometry analysis of cytokines in CD4+ cells. IL-21+ cells with or without PMA and ionomycin stimulation (PMA iono+/-) (gated on TCRβ+ CD4+ cells) (J) Quantification of the frequency of B220+ cells in live cells and absolute number. (K) Flow cytometry analysis of GC-B cells (Fas+ GL-7+ cells) (gated on B220+ cells) in pooled spleen and peripheral LNs (cervical and inguinal) from 14-weeks-old Foxp3Cre WT and Foxp3-Cre Tet2/3fl/fl mice (left). Quantification of the frequency of Fas+ GL-7+ cells in B220+ T cells. [file Image2.tif]

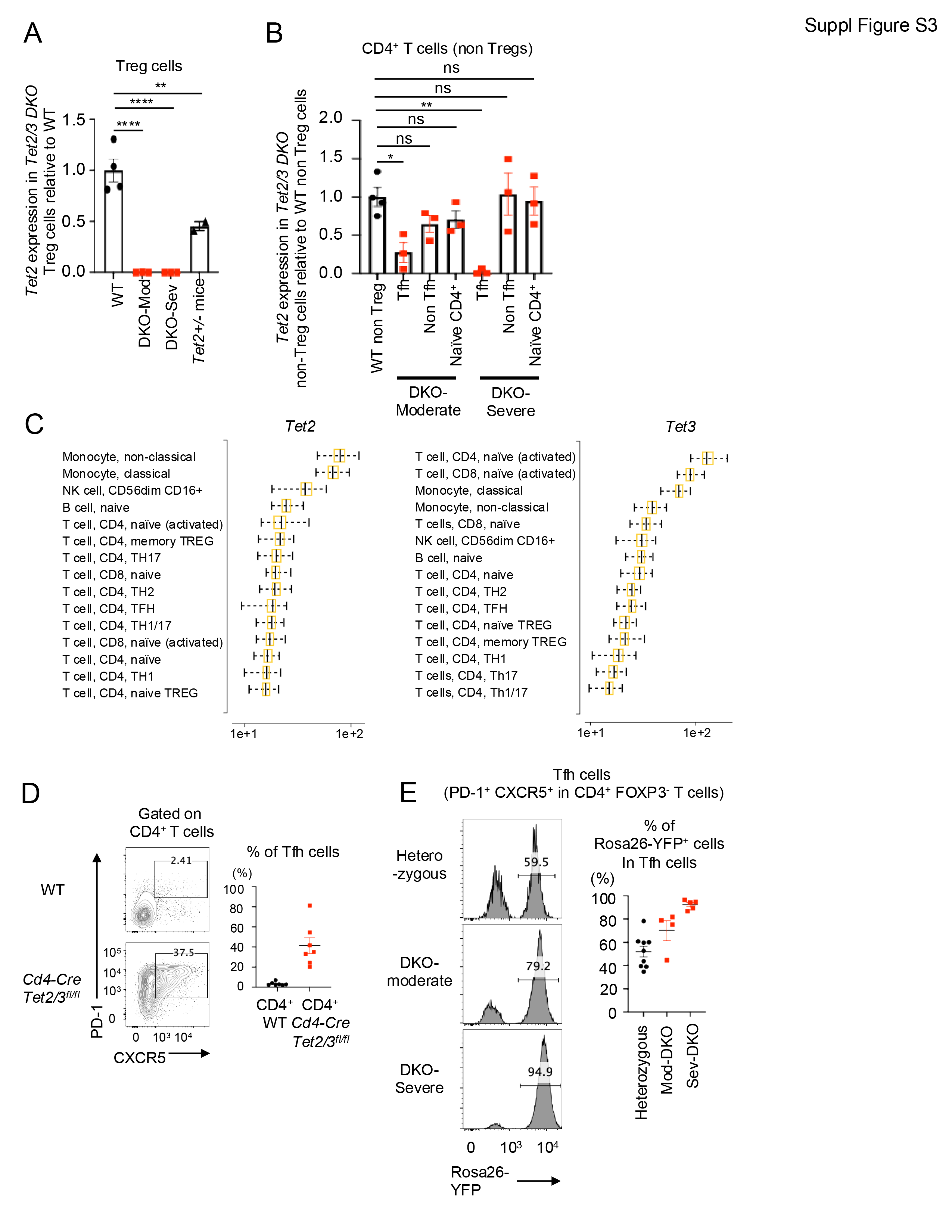

Supplement: Supplementary Figure 3 — (A, B) The graphs show the Tet2 expression in Tet2/3 DKO Treg cells relative to WT Treg cells (A), the Tet2 expression in Tet2/3 DKO non-Treg cells relative to WT non Treg cells (B). Treg cells: CD4+ YFP(FOXP3)+, WT non Treg cells: CD4+ YFP(FOXP3)-, Tfh cells: CD4+ YFP(FOXP3)- PD-1+ CXCR5+, non Tfh cells: CD4+ YFP(FOXP3)- PD-1- CXCR5-, Naïve CD4+ cells: CD4+ YFP(FOXP3)- CD62Lhigh CD44low. (C) Data of Tet2 or Tet3 expression in each cell type from DICE (Database of Immune Cell Expression, Expression quantitative trait loci (eQTLs) and Epigenomics) project. (D) Representative flow cytometry plots of PD-1+ CXCR5+ cells (gated on TCRβ+ CD4+ cells) and quantification of the frequency of PD-1+ CXCR5+ cells in pooled spleen and peripheral lymph nodes (cervical and inguinal) from 4–6 weeks old Tet2/3fl/fl (WT) and Cd4-Cre Tet2/3fl/fl mice. (E) Representative flow cytometry plots of Rosa26-YFP+ cells (gated on PD-1+ CXCR5+ in CD4+ FOXP3- T cells) and quantification of the frequency of the cells in pooled spleen and peripheral lymph nodes (cervical and inguinal) from 14 weeks old Heterozygous (Foxp3-Cre Tet2/3fl/+ Rosa26-YFPLSL), DKO-moderate or DKO-severe Foxp3-Cre Tet2/3fl/fl Rosa26-YFPLSL mice. [file Image3.tif]

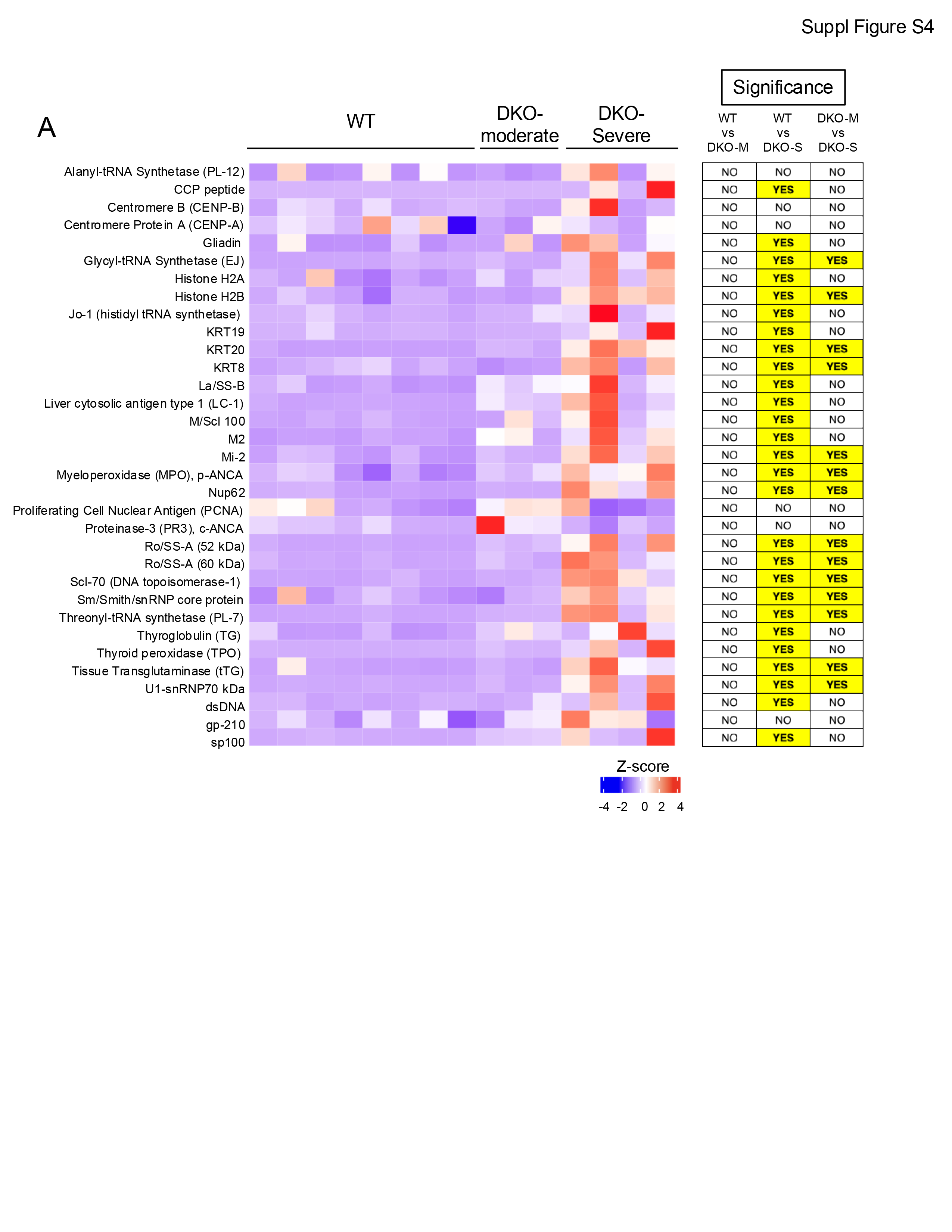

Supplement: Supplementary Figure 4 — Heatmap from polyreactivity assay for serum isolated from WT (n=8) and DKO-moderate (n=3), DKO-severe (n=4) tested by autoantibody array assay kit (13–15 weeks old mice). For data analysis, R package ‘limma’ and multiple comparisons correction was performed (adjusted p value < 0.05 between tested groups). [file Image4.tif]

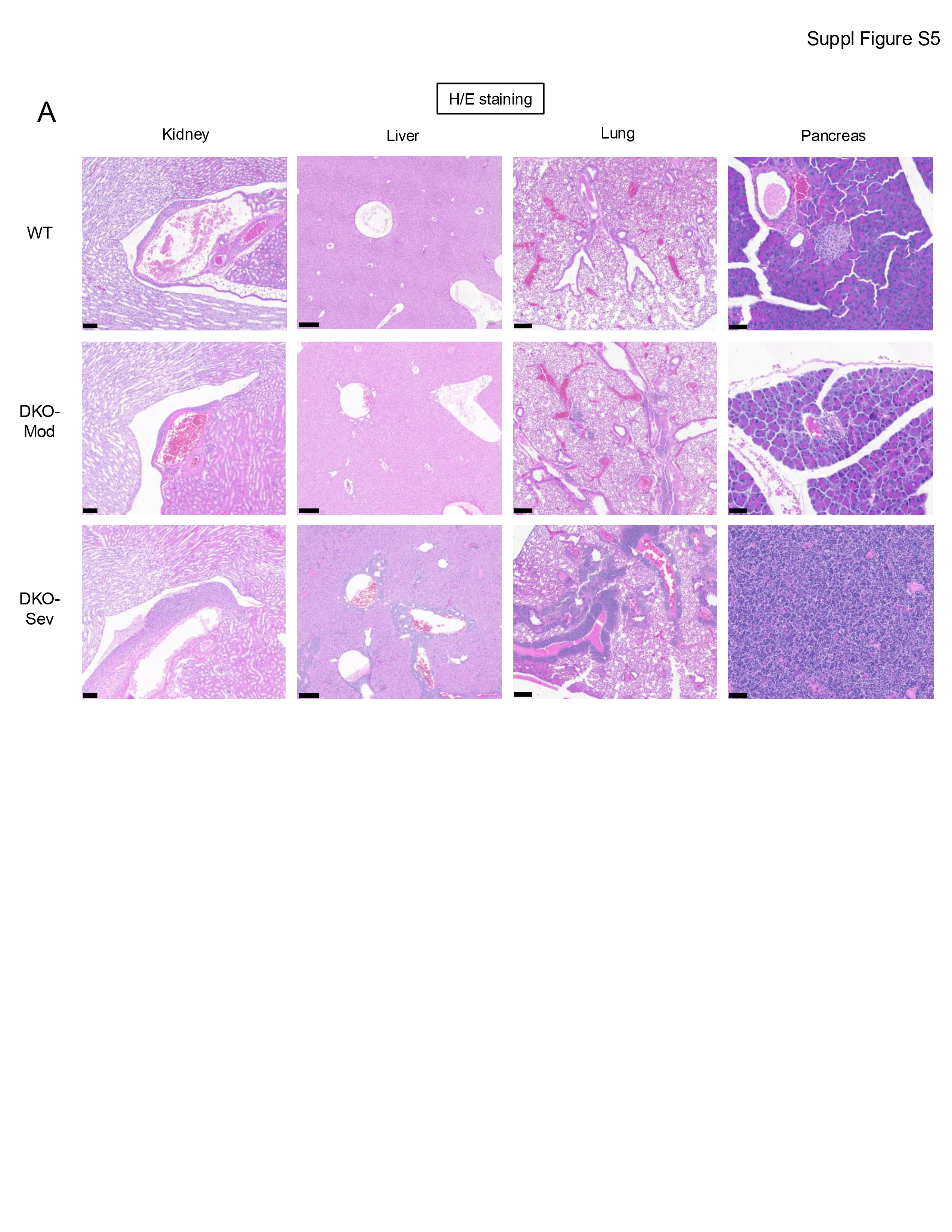

Supplement: Supplementary Figure 5 — H&E staining of Kidney, Liver, Lung and Pancreas from 14-week-old Foxp3Cre WT and Foxp3-Cre Tet2/3fl/fl mice. Scale bar; Kidney: 100μm, Liver: 200μm, Lung: 250μm, Pancreas: 50μm. [file Image5.tif]

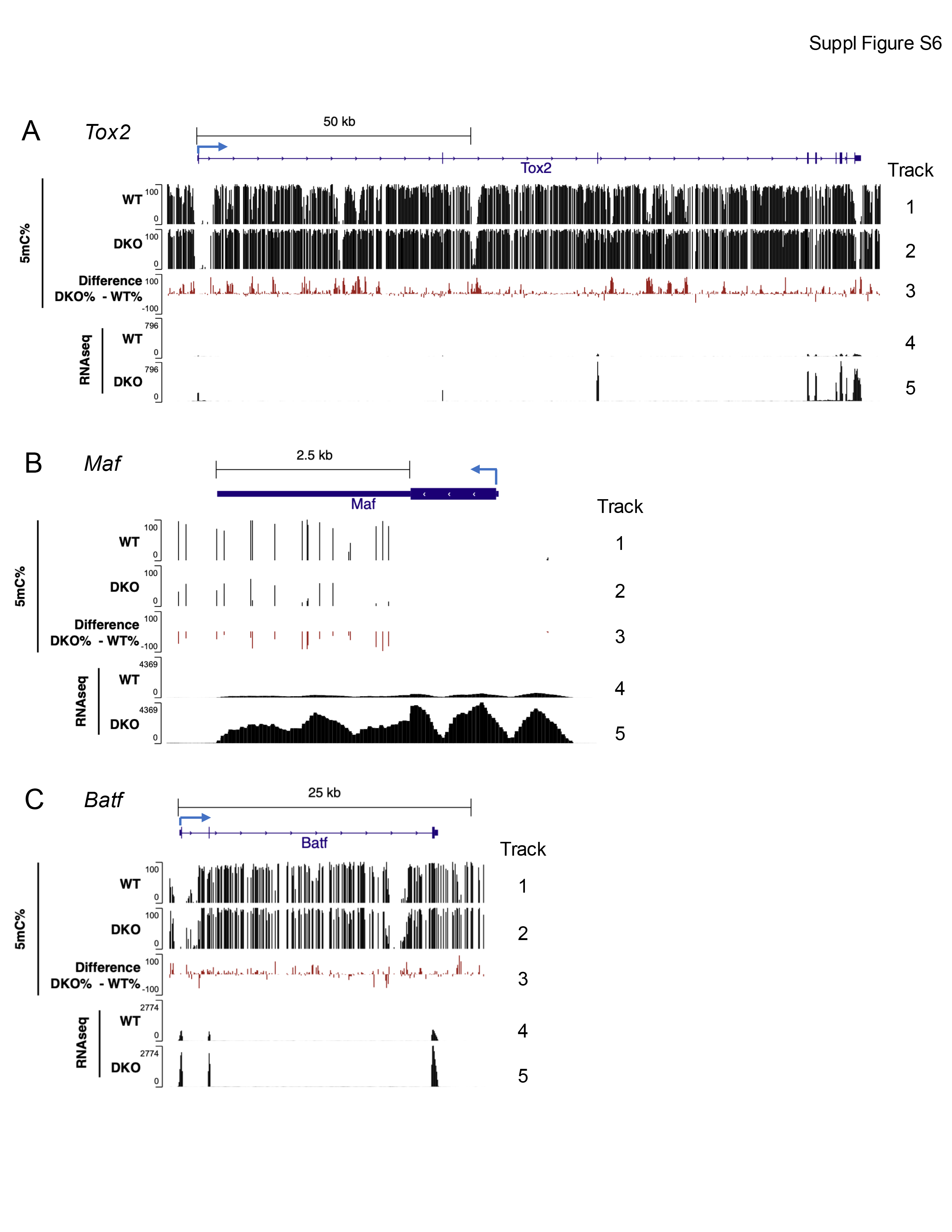

Supplement: Supplementary Figure 6 — (A–C) Genome browser views showing 5mC% (track 1-3) from 6 base sequencing, gene expression (RNA-seq, track 4-5) in Tox2 (A), Maf (B) and Batf (C) locus. 6 base sequencing; WT: naïve CD4+ T cells (CD4+ YFP(FOXP3)- CD62Lhigh CD44low) from Foxp3Cre WT mice, DKO: Tfh like cells (CD4+ YFP(FOXP3)- PD-1+ CXCR5+) from DKO-severe Foxp3-Cre Tet2/3fl/fl mice. RNA seq; WT: CD4+ YFP(FOXP3)- T cells from Foxp3Cre WT mice, DKO: CD4+ YFP(FOXP3)- T cells from DKO-severe Foxp3-Cre Tet2/3fl/fl mice. [file Image6.tif]
